# Structure-Activity Investigations and Optimisations of Non-metabolite Agonists for the Succinate Receptor 1

**DOI:** 10.1038/s41598-018-28263-7

**Published:** 2018-07-03

**Authors:** Elisabeth Rexen Ulven, Mette Trauelsen, Matjaz Brvar, Michael Lückmann, Line Ø. Bielefeldt, Lisa K. I. Jensen, Thue W. Schwartz, Thomas M. Frimurer

**Affiliations:** 1 Department of Physics, Chemistry and Pharmacy, University of Southern Denmark, Campusvej 55, 5230 Odense M, Denmark; 2 NNF Center for Basic Metabolic Research, Section for Metabolic Receptology, Faculty of Health and Medical Sciences, University of Copenhagen, Blegdamsvej 3, 2200 Copenhagen, Denmark; 3 Laboratory for Molecular Pharmacology, Department of Biomedical Research, Faculty of Health and Medical Sciences, University of Copenhagen, Blegdamsvej 3, 2200 Copenhagen, Denmark

## Abstract

The succinate receptor 1 (SUCNR1) is a receptor for the metabolite succinate, which functions as a metabolic stress signal in the liver, kidney, adipose tissue and the retina. However, potent non-metabolite tool compounds are needed to reveal the physiological role and pharmacological potential of SUCNR1. Recently, we published the discovery of a computationally receptor-structure derived non-metabolite SUCNR1 agonist series with high target selectivity. We here report our structure-activity exploration and optimisation that has resulted in the development of agonists with nanomolar potency and excellent solubility and stability properties in a number of *in vitro* assays. Ligand-guided receptor models with high discriminative power between binding of active and inactive compounds were developed for design of novel chemotypes.

## Introduction

The 7-transmembrane G-protein coupled receptor SUCNR1 was first identified in 2001 and found to have close homology with the purinergic receptor P2Y1. The receptor was therefore initially believed to be activated by purinergic compounds^1^. However, in 2004, SUCNR1 was deorphanised and found to be activated by the citric acid cycle intermediate succinate at micromolar concentrations^2^. The other citric acid cycle intermediates oxaloacetate and α-ketoglutarate were also found to activate the receptor, albeit with reduced potency^3,4^. SUCNR1 is highly expressed in liver, kidney, and adipose tissue, but has also been reported in retina, heart, and immune cells^2,5–7^. During oxidative stress succinate can accumulate and reach local concentrations sufficiently high to activate SUCNR1^8^, leading to a variety of unwanted physiological effects such as hypertension^2^, hypertrophy of the heart^7^, inflammation^9^, inhibition of lipolysis in the white adipose tissue^5^, activation of hepatic stellate cells^10^, and vascular growth in the retina^6^.

Although most studies so far indicate that antagonists for SUCNR1 might be optimal from a therapeutic perspective this has yet to be experimentally confirmed and is mainly based on studies using the natural agonist succinate, a compound that beside its relatively weak potency also is an intermediate in the citric acid cycle and exerts effects unrelated to SUCNR1. In 2011, the first and so far only antagonists for SUCNR1 were reported, consisting of a series of naphthyridines that demonstrated good pharmacokinetic properties in rats^11^. Small synthetic succinate analogues have recently been reported as full agonists and although the compounds show improved agonistic potency, the best compound, *cis*-epoxysuccinic acid, has an EC_50_ of only 2.7 μM^3^.

Based on the characterisation of the binding site for succinate in SUCNR1 and identification of an empty side-pocket in the receptor, we have recently published the discovery of novel drug-like SUCNR1 agonists which in accordance with both loss- and gain-of-function mutational data exploited this pocket^4^. Here we report the further development and structure-activity investigations of this agonist series. The generated library of active and inactive compounds together with the original receptor model based on the X-ray structure of the P2Y1 receptor allowed for generation of new SUCNR1 models which could discriminate between active and inactive compounds with high discriminative power. Although the human and mouse receptor orthologues are closely related they are not identical and succinate shows a small increase in potency on the mouse receptor^2^. All compounds have therefore been studied on both the human and mouse orthologues in order to develop tool compounds for further investigations of SUCNR1.

## Results and Discussion

### Synthesis

**Figure 1:**
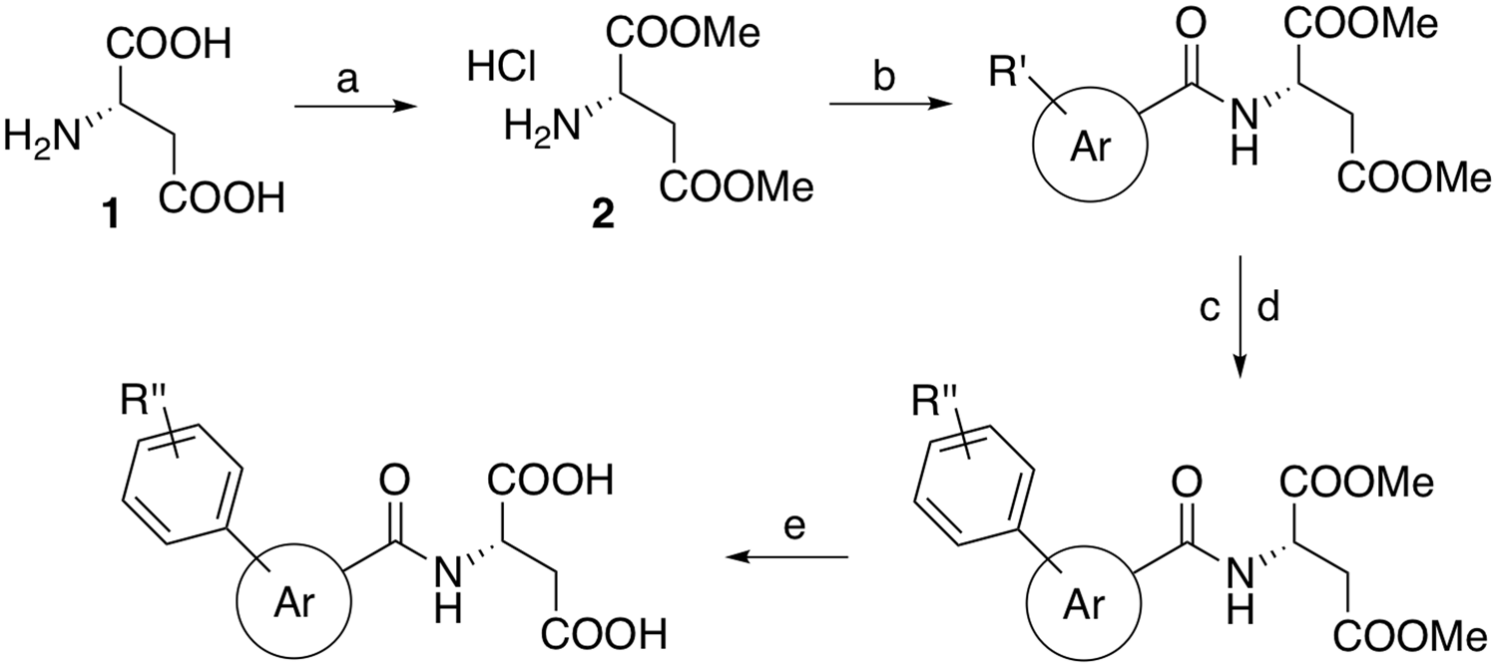
General synthetic route. Reagents and conditions: (**a**) SOCl_2_, MeOH, 0 °C → reflux. (**b**) BTFFH, DIPEA, ArCOOH, DCM, 80 °C. (**c**) ArB(OH)_2_, Pd-XPhos-G4, 0.5 M K_3_PO_4aq_, THF, room temp. (**d**) For R” = OH: K_2_CO_3_, alkylhalide/tosylate, MeCN, 50–55 °C. (**e**) 0.6 M LiOH_aq_, THF, room temp.

All test compounds were synthesised by the same overall route, starting from L-aspartic acid (**1**) that was converted to the corresponding dimethyl ester hydrochloride **2** by an acid catalysed esterification (Fig. 1). Coupling of **2** to various carboxylic acids was efficiently achieved via the corresponding acyl fluoride generated *in situ* by fluoro-*N,N,N’,N’*-bis(tetramethylene)foramidinium hexafluorophosphate (BTFFH)^12^. Halogenated aryl and heteroaryl compounds were coupled with various aryl boronic acids by a Suzuki reaction using the 4th generation Pd-XPhos precatalyst. The phenolic compounds were afterwards alkylated by alkylhalides or –tosylates. Finally, base promoted hydrolysis using LiOH gave the desired dicarboxylic acid test compounds.

### Exploration of structure activity relationship (SAR)

In order to optimise the activity of the lead compound Cmpd (*S*)−130 (**3**), we started by exploring larger modifications of the aromatic part of the molecule (selected examples are included in Table 1). Initially, the aromatic part was truncated by exchanging the fluorophenyl with a bromine (**4**), which resulted in more than one order of magnitude decrease in potency. Exchanging the bromofurane with the halogenated phenyls **5–7** revealed weak activity on both receptor orthologues of the *ortho*-chlorine and *para*-bromine analogs. Only the larger *meta*-iodine substituted compound **6** showed micromolar activity on both receptor orthologues. Inserting a methylene-linker between the amide and the bromophenyl (**8**) resulted in an inactive compound and exchanging the bromine for the fluorophenyl (**9**) only gave a weakly active compound, far less potent than the lead **3**.

**Table 1:** Investigation of miscellaneous amide analogues.


	R	hSUCNR1	mSUCNR1	ClogP^b^
		pEC_50_ (efficacy, %)^a^		
3		5.75 ± 0.08 (72.8 ± 2.6)	6.46 ± 0.06 (79.3 ± 1.6)	1.66
4		4.53 ± 0.13 (78.1 ± 7.5)	4.93 ± 0.05 (95.8 ± 2.9)	0.44
5		22% @10^−4^M	13% @10^−4^M	0.28
6		50% @10^−4^M	64% @10^−4^M	1.52
7		8% @10^−4^M	15% @10^−4^M	1.26
8		5% @10^−4^M	11% @10^−4^M	1.28
9		25% @10^−4^M	15% @10^−4^M	2.44

^a^pEC_50_ values were determined from dose-response curves of induction of IP3 turnover in SUCNR1 transfected HEK cells (N = 3), efficacy is determined relative to succinate (100%). ^b^Calculated by BioByte’s algorithm as implemented in ChemDraw Professional 16.0.1.4 (ClogP option).

Since the smaller compounds did not show sufficient activity we moved the focus to explorations on the terminal phenyl ring (Table 2). Initially, the original 4-fluoro substituent was removed (**10**). This only affected the potency marginally on both receptor orthologues, but exposes a potential metabolically labile site. Next, a methyl scan of the ring was performed to investigate the binding site for additional space (**11**–**13**). Whereas both the *ortho*- and *meta*-methyl were tolerated only the *para*-methyl **13** led to a more potent compound on both human and mouse SUCNR1. Attempts to pick up hydrogen bond interactions revealed that 2-methoxy (**16**) was equivalent to the 2-methyl in potency and that 3-hydroxymethyl (**14**) was less tolerated than the 3-methyl (**12**) with a 3-fold decrease in potency. Again, the *para*-position was favoured with the 4-hydroxy (**15**) being almost equipotent with the 4-methyl compound. Further exploration of the *para*-position indicated that this part of the binding pocket is able to accommodate polar substituents such as methoxy (**17**) and nitrile (**19**) as well as nonpolar substituents such as trifluoromethyl (**18**), all showing similar potencies in the submicromolar range on the human receptor orthologue and with **17** slightly favoured on the mouse orthologue.

**Table 2:** Investigation of ring A.


	Ring A	hSUCNR1	mSUCNR1	ClogP^b^
		pEC_50_ (efficacy, %)^a^		
3^*c*^		5.75 ± 0.08 (72.8 ± 2.6)	6.46 ± 0.06 (79.3 ± 1.6)	1.66
10		5.29 ± 0.19 (81.6 ± 8.4)	6.16 ± 0.11 (81.3 ± 3.6)	1.47
11		5.27 ± 0.18 (94.7 ± 8.7)	6.13 ± 0.10 (76.7 ± 3.1)	1.67
12		5.02 ± 0.10 (91.9 ± 5.3)	6.07 ± 0.09 (81.6 ± 3.0)	1.97
13		6.33 ± 0.14 (69.1 ± 3.9)	6.83 ± 0.11 (73.2 ± 2.7)	1.97
14		4.81 ± 0.20 (74.2 ± 11.8)	5.71 ± 0.09 (79.6 ± 3.7)	0.43
15		6.13 ± 0.14 (71.1 ± 4.2)	6.82 ± 0.08 (74.8 ± 2.0)	0.87
16		5.29 ± 0.15 (103.8 ± 7.9)	6.14 ± 0.10 (78.1 ± 3.1)	0.89
17		6.41 ± 0.14 (85.3 ± 4.5)	7.39 ± 0.07 (84.2 ± 1.8)	1.45
18		6.55 ± 0.25 (81.0 ± 7.2)	6.81 ± 0.17 (80.6 ± 4.6)	2.43
19		6.39 ± 0.11 (72.1 ± 3.2)	6.79 ± 0.06 (76.0 ± 1.5)	1.00

^a^pEC_50_ values were determined from dose-response curves of induction of IP3 turnover in SUCNR1 transfected HEK cells (N = 3), efficacy is determined relative to succinate (100%). ^b^Calculated by BioByte’s algorithm as implemented in ChemDraw Professional 16.0.1.4 (ClogP option). ^c^Duplication of data from Table 1.

To further explore the 4-methoxy compound, the most potent analogue on the mouse receptor, a sub series of *para*-alkoxy analogues were investigated (Table 3). The larger and more electronegative trifluoromethoxy (**20**) showed a 2-fold increase on hSUCNR1 but was accompanied by >4-fold decrease on mSUCNR1, rendering the compound approximately equipotent on both orthologues. Expanding to either ethoxy (**21**) or *i*-propoxy (**22**) resulted in a small increase in potency, indicating that the binding pocket can accommodate more elongated and bulky substituents. To explore whether or not the binding site could also accommodate larger hydrophilic substituents the oxatane **23** was investigated and found tolerable, but led to >2-fold reduction of potency. Finally, the mesylpropoxy analogue **24** was explored. This appendage, which lowers the lipophilicity an order of magnitude, has previously been applied to lower lipophilicity of ligands for the free fatty acid receptor FFA1 and has proven to be a metabolically stable substituent^13,14^. Lipophilicity of this compound is in the low end of the desired range but it is interesting to note that the compound exhibited a ligand lipophilicity efficiency (LLE) >5 based on ClogP and almost sustained potency on both mSUCNR1 and hSUCNR1.

**Table 3:** Alkoxy analogues.


	R	hSUCNR1	mSUCNR1	ClogP^b^
		pEC_50_ (efficacy, %)^a^		
17^*c*^	Me	6.41 ± 0.14 (85.3 ± 4.5)	7.39 ± 0.07 (84.2 ± 1.8)	1.45
20	F_3_C	6.99 ± 0.15 (61.7 ± 3.6)	6.72 ± 0.11 (69.7 ± 2.8)	3.17
21	Et	6.59 ± 0.14 (56.3 ± 3.1)	7.44 ± 0.09 (70.2 ± 2.0)	1.98
22	*i*Pr	6.84 ± 0.14 (69.6 ± 4.3)	7.29 ± 0.07 (75.6 ± 1.7)	2.29
23		6.27 ± 0.11 (76.5 ± 3.1)	6.97 ± 0.07 (82.4 ± 1.9)	1.81
24		6.10 ± 0.09 (63.9 ± 2.4)	7.33 ± 0.09 (74.6 ± 2.1)	0.53

^a^pEC_50_ values were determined from dose-response curves of induction of IP3 turnover in SUCNR1 transfected HEK cells (N = 3), efficacy is determined relative to succinate (100%). ^b^Calculated by BioByte’s algorithm as implemented in ChemDraw Professional 16.0.1.4 (ClogP option). ^c^Duplication of data from Table 2.

Subsequently, the attention was directed towards the central aromatic ring (Table 4). Replacing the furane of **3** with the 1,3-substituted phenyl **25** improved the potency somewhat on the human orthologue whereas the potency on the mouse orthologue decreased 10-fold. The 1,4-substituted phenyl **26** clearly led to an unfavoured geometry with only trace activity on SUCNR1. The corresponding 2,6-substituted pyridine **27** sustained the potency on hSUCNR1 and almost regained the potency on mSUCNR1.

**Table 4:** Investigation of ring B.


	Ring B	hSUCNR1	mSUCNR1	ClogP^b^
		pEC_50_ (efficacy, %)^a^		
3^*c*^		5.75 ± 0.08 (72.8 ± 2.6)	6.46 ± 0.06 (79.3 ± 1.6)	1.66
25		5.91 ± 0.11 (49.7 ± 3.9)	5.40 ± 0.13 (58.0 ± 4.5)	2.27
26		4% @10^−4^M	7% @10^−4^M	2.27
27		6.03 ± 0.25 (78.2 ± 8.0)	6.10 ± 0.17 (89.3 ± 6.1)	2.05

^a^pEC_50_ values were determined from dose-response curves of induction of IP3 turnover in SUCNR1 transfected HEK cells (N = 3), efficacy is determined relative to succinate (100%). ^b^Calculated by BioByte’s algorithm as implemented in ChemDraw Professional 16.0.1.4 (ClogP option). ^*c*^Duplication of data from Table 1.

Next, a small selection of compounds was synthesised to combine the observed SAR of the alkoxy-substituents and alterations of the central ring (Table 5). The 4-methoxy and 4-trifluoromethoxy substituents, being the most potent alkoxy-substituents on the mouse and human receptor orthologues, respectively, were attached to compounds bearing the 1,3-substituted phenyl and the 2,6-substituted pyridine as the central ring. No improvement was observed for the methoxy analogues (**28**–**29**). In contrast, the trifluoromethoxy analogues (**30–31**) were found to be the most potent compounds on hSUCNR1, but unfortunately without high potency on mSUCNR1, especially for **30**. The elongated trifluoroethoxy analogue **32** showed improved potency on the mouse orthologue. Still, **31** remained the most potent agonist on hSUCNR1. Finally, the 4-propoxy analogue **33** was found to be approximately twice as potent as the corresponding methoxy analogue **29** and almost equipotent on both orthologues.

**Table 5:** Combined analogues.


	Ring A	Ring B	hSUCNR1	mSUCNR1	ClogP^b^
			pEC_50_ (efficacy, %)^a^		
28			6.79 ± 0.12 (58.0 ± 2.7)	5.92 ± 0.09 (64.0 ± 2.5)	2.01
29			6.85 ± 0.19 (52.6 ± 3.9)	6.74 ± 0.13 (71.2 ± 3.2)	1.07
30			7.23 ± 0.14 (49.2 ± 2.7)	5.84 ± 0.16 (45.3 ± 3.4)	3.11
31			7.64 ± 0.13 (61.7 ± 2.5)	6.75 ± 0.09 (69.9 ± 2.2)	2.18
32			7.23 ± 0.23 (88.7 ± 6.1)	7.00 ± 0.09 (68.1 ± 2.5)	1.86
33			7.23 ± 0.37 (66.4 ± 7.2)	7.29 ± 0.18 (61.5 ± 3.8)	2.13

^a^pEC_50_ values were determined from dose-response curves of induction of IP3 turnover in SUCNR1 transfected HEK cells (N = 3), efficacy is determined relative to succinate (100%). ^b^Calculated by BioByte’s algorithm as implemented in ChemDraw Professional 16.0.1.4 (ClogP option).

**Figure 2:**
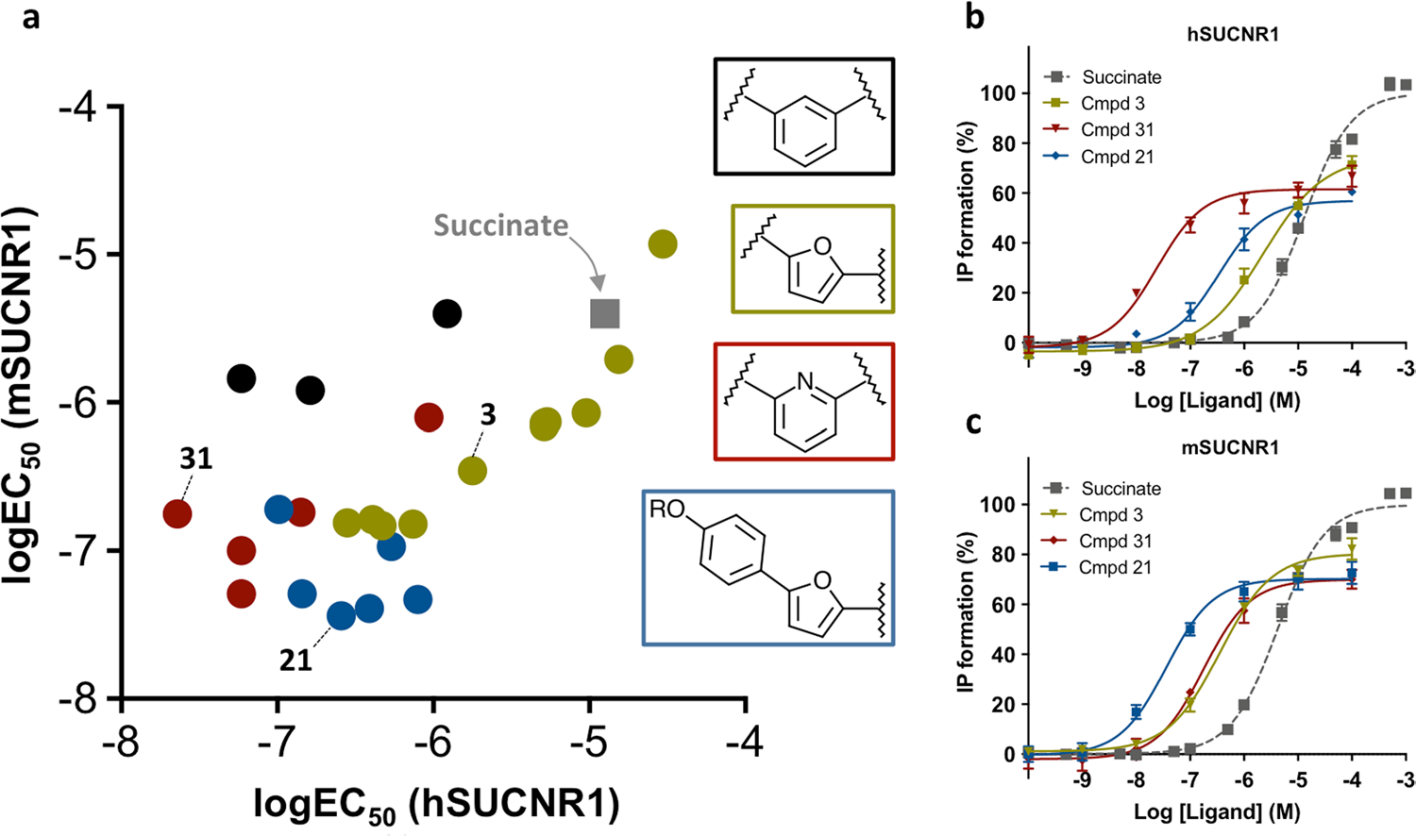
Summary of *in vitro* agonist potencies. (**a**) Scatterplot of *in vitro* agonist potencies by IP3 accumulation in SUCNR1 transfected HEK-293 cells of the compounds in Tables 1–5 on the hSUCNR1 and mSUCNR1 receptor. (**b**,**c**) Dose-response curves for succinate and selected compounds (**3**, **21** and **31**) on hSUCNR1 and mSUCNR1.

To get a better overview of the observed SAR on the human and mouse receptor orthologues a scatterplot of all active compounds was made (Fig. 2a). The compounds were color-coded according to the central or terminal ring, which clearly indicated that compounds with a central *meta*-substituted phenyl were better tolerated on hSUCNR1. The 2,6-substituted pyridines were in general more potent and especially **31** was favoured on hSUCNR1. Compounds with a furane as central ring were in general equipotent on the two receptor orthologues, but with addition of a phenalkoxy moiety, the compounds became more potent on mSUCNR1 with **21** being the most potent. Furthermore, dose-response curves of succinate, **3**, **21**, and **31** clearly showed that the non-metabolite compounds were partial agonists, with clear species differences on potency but not efficacy (Fig. 2b,c). The partial agonism was observed for all agonistic compounds (Tables 1–5, Supplementary Fig. S1).

### Molecular modelling and unbiased ligand guided refinement of SUCNR1 receptor-ligand complexes

To describe the detailed molecular mechanism of action and to rationalise the structure-activity data, we docked the compounds in Tables 1–5 to a model of SUCNR1 in complex with the lead compound (**3**) supported by a dozen of site directed mutagenesis data as presented earlier^4^. Despite that we observed binding conformations similar to the lead and with favourable scores, the model - as perhaps could be expected - was unable to rank the compounds in reasonable agreement with the activity data. The interpretation of SAR and structure based design reaches its maximum potential when the receptor displays the structural changes needed for ligand binding, as it has previously been shown in a blind prediction assessment of adenosine A2a receptor complexes (GPCR Dock 2008)^15–18^. We therefore applied an iterative Automated Ligand-guided Backbone Ensemble Receptor Optimisation protocol (ALiBERO)^19^, which samples full receptor and ligand flexibility guided by the ligand information gained in this study to validate and build confidence in the model.

In brief, starting from the homology model supported by mutagenesis data^4^, ALiBERO introduced receptor flexibility via Normal Mode Analysis and Monte Carlo sampling, to generate a small subset of receptor models (pockets). All compounds tested in this study were grouped into an active (EC_50_ ≤ 10 µM) and a decoy set (EC_50_ > 10 µM) consisting of 25 compounds each. For a list of all compounds used in the optimisation protocol, see Table S1. Receptor structures were then chosen based on their ability to discriminate actives from inactives in a retrospective virtual screening using the docking protocol and scoring function in ICM (Molsoft L.L.C., San Diego, CA, USA)^20^, as measured by the normalised square root area under the curve (NSQ_AUC). The best-performing structures from the first generation were consequently selected for the next generation and the steps were repeated in an iterative fashion until maximum docking performance of receptor structures to enrich active compounds was reached. The ALiBERO-optimised receptor ensemble was subsequently validated in a virtual ligand screening using an external test set with a higher active:decoy ratio (~1:50).

**Figure 3:**
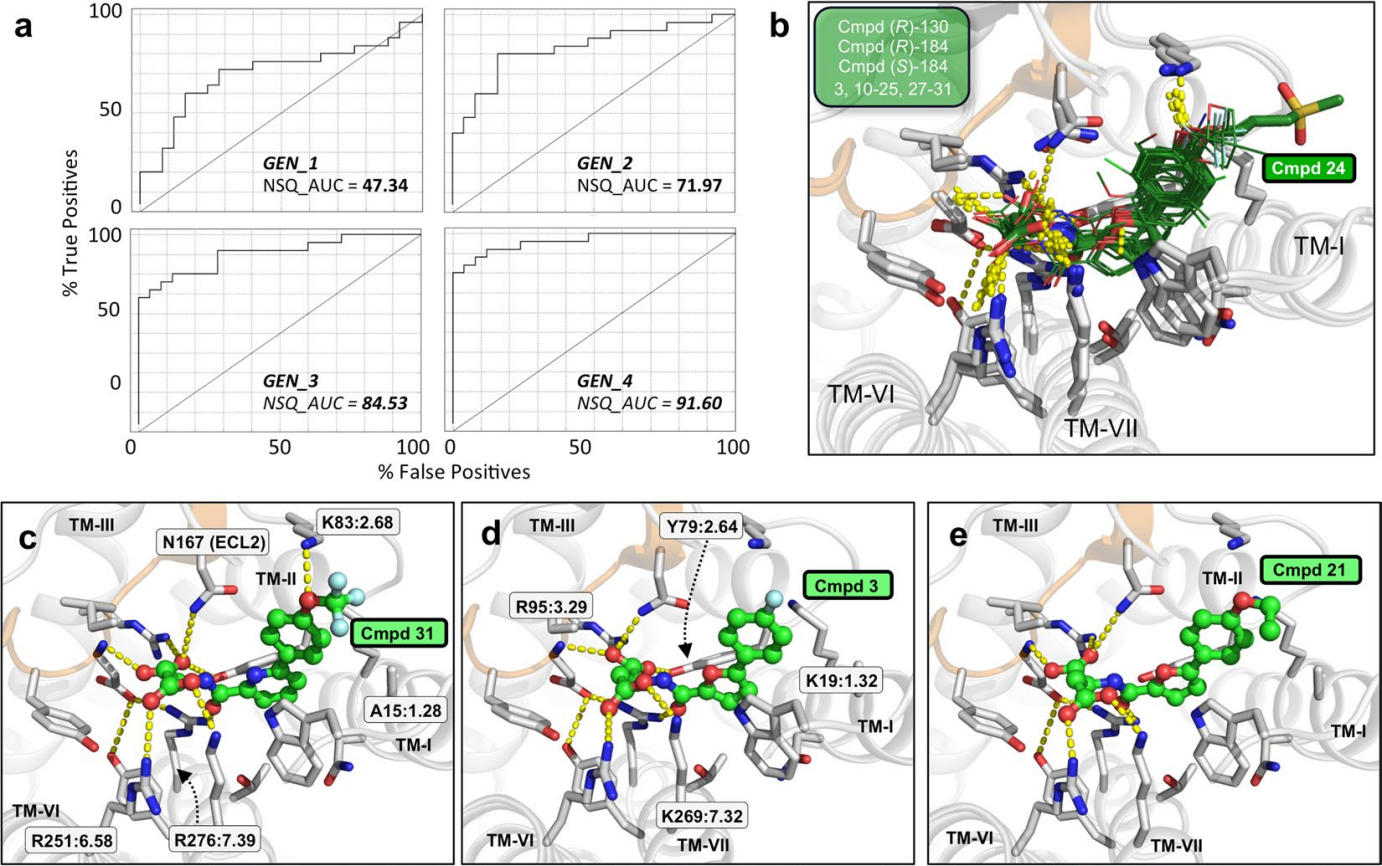
Iterative SUCNR1 receptor optimisation based on ligand information gained in this study. (**a**) Small-scale virtual ligand screening (VLS) results at different stages of the optimisation process (generations GEN_1 - GEN_4). Receiver operating characteristic (ROC) curves and normalised square root area under the curve (NSQ_AUC) values are shown for each generation of receptor pockets. The diagonal corresponds to a random VLS performance. (**b**) Best-performing receptor pocket ensemble (GEN_4), consisting of three receptor structures shown with the best-scored docking poses of all 25 active compounds investigated in this study (dark green lines). Best-scored docking poses of compound **24** (**b**), **31** (**c**), **3** (**d**) and **21** (**e**) are shown in green sticks. Polar receptor-ligand interactions are indicated by yellow spheres. Homology models are based on the x-ray crystal structure of the P2Y_1_ receptor (PDB 4XNW) which shares several key side-chains in the binding site with SUCNR1, i.e. Y79:2.64, D174 and R276:7.39. Figure was made using PyMol.

Notably, the ligand-guided ALiBERO-based mSUCNR1 models demonstrated a dramatic improvement in retrospective virtual screening performance of the developed compounds compared with the initial homology models and proved successful in separating the majority of active from inactive ligands in docking screens (Fig. 3a, Supplementary Fig. S2). It is interesting to note that the active ligands bind to an extended binding cavity in a very consistent pose compared to the lead compound (**3**) supported by both loss-of-function but also gain-of-function mutagenesis data as we have reported previously^4^. For example, the receptor mutants R251:6.58 L and R276:7.39 F showed a potency decrease of more than 100-fold. The binding cavity of SUCNR1 is characterised by a polar network consisting of residues in TM-II (Y79:2.64), TM-III (R95:3.29), TM-VI (R248:6.55, R251:6.58), TM-VII (K269:7.32, Y272:7.35, R276:7.39), ECL2 (D174) as well as a relatively hydrophobic subpocket spanning between TM-I, -II, ECL1 and −2 up towards the extracellular surface of the receptor (residue numbering according to gpcrdb.org). The optimised receptor ensemble (Fig. 3b) accommodates the active compounds in a common binding mode in which the left-hand side carboxylic acids make hydrogen bond interactions with R95:3.29, R251:6.58 and R276:7.39 and where the right-hand side of the ligands adopts an angled conformation that is defined by the linker between ring A and B in good agreement with the observed structure-activity relationship. Due to the shape and direction of the subpocket accommodating ring A, compounds with a bent conformation between the amide and the terminal ring, e.g. compounds having 2,5-substitued furan or *meta*-substituted phenyl as central ring, are sterically better tolerated than e.g. the *para*-substituted phenyl **26** and elongated *para*-substituted phenyl **9**. Furthermore, compounds with a pyridine (e.g. **27**, **29**, **31**), or furan as central ring, can stabilise the favoured bent conformation by intramolecular hydrogen bonding between the pyridine nitrogen or furan oxygen and the amide N-H, thereby inducing an optimal low energy conformation.

The subpocket that is occupied by ring A, contains W10:1.42 which allows for parallel displaced aromatic stacking interactions that are present in all binding poses for the active ligands. As this pocket spans all the way to the extracellular tips of TM-I and -II, it is able to accomodate longer substituents, such as the mesylate **24** (Fig. 3b), which likely interacts with solvent water molecules on the extracellular surface of the receptor cavity. Compounds that contain *O*-alkyl, hydroxyl, or nitrile groups in the *para*-position on ring A, such as **15**, **17**, **19–24** and **28–31**, can form hydrogen bond interactions with K83 (Asn87 in hSUCNR1) and backbone NH of Glu170 in ECL1 and −2b (Fig. 3c). Due to the geometry of this interaction, *ortho*- and *meta*-subtitutions (**14** + **16**) are less favorable.

While the polar residues that coordinate the dicarbocylic acid moiety are highly conserved between mouse and human SUCNR1, the hydrophobic subpocket accommodating ring A is more diverse with several differences in ECL1 (Lys83/Asn87), ECL2 (Lys162/Thr166, Glu163/Asp166, Glu164/Asn166, Asn167/Thr171) and TM-VII (Lys269/Asn274) between mSUCNR1 and hSUCNR1, respectively. The Lys83/Asn87 difference between mSUCNR1 and hSUCNR1 might explain the higher potencies of the trifluoromethoxy-substituted compounds **30** and **31** on hSUCNR1, as these bulky moities likely form more favorable interactions with the shorter Asn side-chain (Fig. 3c).

In conclusion, the developed ligand-guided SUCNR1 models are consistent with the SAR, and they are sufficiently accurate to separate actives from decoys, suggesting that the models will be valuable in prospective studies to support structure-based drug design of additional chemotypes directly related to drug discovery applications.

### Physicochemical and *in vitro* stability properties

Based on the SAR exploration, the original lead (**3**) and the most potent agonists on hSUCNR1 (**31**) and mSUCNR1 (**21**) were selected for further property investigations. All compounds showed excellent chemical stability (>98% after 70 hours) and kinetic solubility (>200 μM) in 10 mM PBS_7.4_. However, the high aqueous solubility made logD_7.4_ determinations very challenging and only the most lipophilic compound **31** could be quantified (logD_7.4_ = −2.13). The stability of the compounds was further examined in a selection of simulated gastrointestinal fluids (FaSSGF, FaSSIF and FeSSIF) and all compounds were found to be stable for 2 hours, with exception of **31** in FeSSIF, which could not be determined due to overlapping UV-absorption of the compound and media. Finally, the stability in mouse liver microsomes was investigated and all compounds were found to be stable for 1 hour, possibly partly due to the hydrophilic nature of the compounds.

## Conclusion

We here report the structure-activity investigations of a series of non-metabolite SUCNR1 agonists that was originally identified from a computational, receptor-structure derived agonistic lead. ALiBERO optimised homology models of the mouse SUCNR1 were developed based on the x-ray structure of the closely related P2Y1 receptor and found to be sufficiently accurate to discriminate between actives and inactives and could explain the majority of the SAR observations. The exploration led to development of potent drug-like, non-metabolite tool compounds with nanomolar potency on both the human and murine receptor orthologues and excellent physicochemical and *in vitro* stability properties. We believe these compounds will be useful for further investigations of SUCNR1 as a potential therapeutic target and pharmacokinetic studies in mice are currently ongoing and will reveal if absorption of the compounds might be challenged by their hydrophilic nature and if the excellent stability properties are conserved *in vivo*.

## Methods

### General

Commercial starting materials and solvents were used without further purification, unless otherwise stated. THF was freshly distilled from sodium/benzophenone. DCM was distilled and stored over 3 Å sieves. MeCN and *N,N*-diisopropylethylamine were dried over 3 Å sieves. K_2_CO_3_ was dried and stored in an oven. TLC was performed on TLC silica gel 60 F_254_ plates and visualised at 254 or 365 nm or by staining with phosphomolybdic acid, ninhydrin, or KMnO_4_ stains. Purification by flash chromatography was carried out using silica gel 60 (0.040–0.063 mm, Merck). ^1^H and ^13^C NMR spectra were recorded at 400 and 101 MHz, respectively, on a Bruker Avance III 400 at 300 K. Spectra were calibrated relative to the internal standard TMS or residual solvent peak: CDCl_3_ (δ_C_ = 77.16 ppm, δ_H_ = 7.26 ppm), DMSO-*d*_*6*_ (δ_C_ = 39.52 ppm, δ_H_ = 2.50 ppm) and acetone-*d*_*6*_ (δ_C_ = 29.84 ppm, δ_H_ = 2.05 ppm).

HPLC analysis was performed using a Gemini C18 column (5 μm, 4.6 × 150 mm); flow: 1 mL/min; 10% MeCN in water (0–1 min), 10–100% MeCN in water (1–10 min), 100% MeCN (11–15 min), with both solvents containing 0.1% HCOOH as modifier; UV detection at 254 nm (or 280 nm, 304 nm and 315 nm depending on test compound absorption maximum for solubility and stability studies). UPLC analysis was performed using a 100 Å C18 column (1.7 μm, 2.1 × 100 mm); flow: 0.3 mL/min; 70% MeOH in water (0–6 min), the water containing 0.1% HCOOH as modifier; UV detection at 254 nm, 280 nm, 304 nm or 315 nm depending on test compound absorption maximum. High-resolution mass spectra (HRMS) were obtained on a Bruker micrOTOF-Q II (ESI). Optical rotation was measured on an Anton Paar MCP 100 Polarimeter (Anton Paar Cell 100 mm, CL. 0.01, Ø 5 mm). Purity was determined by HPLC (254 nm) and confirmed by inspection of NMR spectra. The purity of all test compounds were >95%.

### Amide coupling

An oven dried microwave vial under argon atmosphere was charged with the acid (1.3 equiv), dry DCM (2 mL/mmol), *N,N*-diisopropylethylamine (5.5 equiv) and BTFFH (1.5 equiv). The reaction mixture was stirred at rt for 30 min before the HCl salt of the amine (1 equiv) was added. After addition, the vial was capped and heated to 80 °C overnight. The reaction was cooled to rt and diluted with water and extracted with EtOAc (x3). The organic phases were combined, washed with brine, dried over Na_2_SO_4_ and concentrated *in vacuo*. The residue was purified by flash column chromatography (SiO_2_, EtOAc:petroleum ether).

### Suzuki coupling

A schlenck flask under argon was charged with boronic acid (1.1 equiv), aryl/pyridyl halide (1 equiv) and Pd-XPhos-G4 (2 mol%). The flask was evacuated and backfilled with argon (x3). THF (5 mL/mmol) and aqueous 0.5 M K_3_PO_4_ (2 equiv) was added, and the reaction was stirred at rt. After completion, the reaction mixture was diluted with water and extracted with EtOAc (x3). The organic phases were combined, washed with brine, dried over Na_2_SO_4_ and concentrated *in vacuo*. The residue was purified by flash column chromatography (SiO_2_, EtOAc:petroleum ether).

### Alkylation

The phenol (1 equiv) was dissolved in dry MeCN (~6 mL/mmol) in a dry vial under argon atmosphere. The alkyl halide/tosylate (2–7 equiv) and dry K_2_CO_3_ (2 equiv) were added and the reaction was stirred at 50–55 °C until consumption of the phenol as monitored by TLC. After completion, the reaction was diluted with water and extracted with EtOAc (x3). The organic phases were combined, washed with brine, dried over Na_2_SO_4_, and concentrated *in vacuo*. The residue was purified by flash column chromatography (SiO_2_, EtOAc:petroleum ether).

### Ester hydrolysis

The ester (1 equiv) was dissolved in THF (~6 mL/mmol), and aqueous 0.6 M LiOH (3 equiv) was added. The reaction was stirred at rt until consumption of the ester as monitored by TLC. After completion, the reaction was diluted with water, acidified with aqueous 1 M HCl and extracted with EtOAc (x3). The organic phases were combined, washed with brine, and dried over Na_2_SO_4_. The residue was concentrated *in vacuo* to give the pure title compounds.

### (5-Bromofuran-2-carbonyl)-*L*-aspartic acid (4)

Dimethyl (5-bromofuran-2-carbonyl)-*L*-aspartate (**4e**) was synthesised from 5-bromo furoic acid (126 mg, 0.66 mmol) and **2** (100 mg, 0.51 mmol) according to the general amide coupling procedure. Purification by flash column chromatography (EtOAc:petroleum ether, 1:2) gave 136 mg (81%) of a yellow oil: Rf = 0.30 (EtOAc:petroleum ether, 1:1); ^1^H NMR (400 MHz, CDCl_3_) δ 7.29 (d, *J* = 7.9 Hz, 1H), 7.09 (d, *J* = 2.5 Hz, 1H), 6.45 (d, *J* = 3.5 Hz, 1H), 5.02 (dt, *J* = 8.6, 4.5 Hz, 1H), 3.80 (s, 3H), 3.73 (s, 3H), 3.13 (dd, *J* = 17.3, 4.5 Hz, 1H), 2.94 (dd, *J* = 17.3, 4.5 Hz, 1H); ^13^C NMR (101 MHz, CDCl_3_) δ 171.4, 170.8, 156.8, 148.9, 125.1, 117.3, 114.2, 53.0, 52.1, 48.3, 36.1; ESI-HRMS: calcd for C_11_H_12_BrNNaO_6_ (M + Na)^+^ 355.9740, found 355.9757; [α]^20^_D_ − 16.5° (*c* 0.2, MeOH).

**4** was synthesised from **4e** (35 mg, 0.10 mmol) according to the general ester hydrolysis procedure to give 28 mg (88%) of a white solid: *t*_*R*_ = 7.68 min (HPLC); ^1^H NMR (400 MHz, Acetone-*d*_*6*_) δ 7.91 (d, *J* = 7.9 Hz, 1H), 7.13 (d, *J* = 3.5 Hz, 1H), 6.67 (d, *J* = 3.5 Hz, 1H), 5.02–4.94 (m, 1H), 3.02 (d, *J* = 5.8 Hz, 2H); ^13^C NMR (101 MHz, Acetone-*d*_*6*_) δ 172.4, 172.3, 157.4, 150.7, 125.2, 117.4, 115.1, 49.4, 36.4; ESI-HRMS calcd for C_8_H_9_BrNNaO_6_ (M + Na)^+^ 327.9427, found 327.9431; [α]^20^_D_ + 7.2° (*c* 0.2, MeOH).

### (2-Chlorobenzoyl)-*L*-aspartic acid (5)

Dimethyl (2-chlorobenzoyl)-*L*-aspartate (**5e**) was synthesised from 2-chlorobenzoic acid (103 mg, 0.66 mmol) and **2** (99 mg, 0.50 mmol) according to the general amide coupling procedure. Purification by flash column chromatography (EtOAc:petroleum ether, 1:1) gave 121 mg (80%) of a pale yellow solid: Rf = 0.65 (EtOAc); ^1^H NMR (400 MHz, CDCl_3_) δ 7.70–7.66 (m, 1H), 7.44–7.30 (m, 3H), 7.25–7.23 (m, 1H), 5.11–5.05 (m, 1H), 3.80 (s, 3H), 3.70 (s, 3H), 3.19–2.98 (m, 2H); ^13^C NMR (101 MHz, CDCl_3_) δ 171.6, 171.0, 166.2, 134.4, 131.8, 131.2, 130.5, 130.4, 127.2, 53.1, 52.2, 49.3, 36.2; ESI-HRMS calcd for C_13_H_14_ClNNaO_5_ (M + Na)^+^ 322.0453, found 322.0461; [α]^20^_D_ − 22.5° (*c* 0.2, MeOH).

**5** was synthesised from **5e** (25 mg, 0.08 mmol) according to the general ester hydrolysis procedure to give 22 mg (97%) of a white solid: *t*_*R*_ = 7.34 min (HPLC); ^1^H NMR (400 MHz, Acetone-*d*_*6*_) δ 10.80 (br s, 2H), 7.88–7.80 (m, 1H), 7.60–7.55 (m, 1H), 7.50–7.44 (m, 2H), 7.44–7.36 (m, 1H), 5.08–4.98 (m, 1H), 3.09–2.96 (m, 2H); ^13^C NMR (101 MHz, Acetone-*d*_*6*_) δ 172.3, 172.2, 166.9, 136.8, 132.1, 131.6, 130.8, 130.4, 127.9, 50.0, 36.4; ESI-HRMS calcd for C_11_H_9_ClNO_5_ (M − H)^−^ 270.0175, found 270.0177; [α]^20^_D_ + 2.0° (*c* 0.2, MeOH).

### (3-Iodobenzoyl)-*L*-aspartic acid (6)

Dimethyl (3-iodobenzoyl)-*L*-aspartate (**6e**) was synthesised from 3-iodobenzoic acid (161 mg, 0.65 mmol) and **2** (99 mg, 0.50 mmol) according to the general amide coupling procedure. Purification by flash column chromatography (EtOAc:petroleum ether, 1:1) gave 164 mg (84%) of a pale yellow sticky oil: Rf = 0.33 (EtOAc:petroleum ether, 1:1); ^1^H NMR (400 MHz, CDCl_3_) δ 8.17–8.12 (m, 1H), 7.88–7.83 (m, 1H), 7.78–7.72 (m, 1H), 7.22–7.14 (m, 2H), 5.08–4.98 (m, 1H), 3.80 (s, 3H), 3.71 (s, 3H), 3.20–2.91 (m, 2H); ^13^C NMR (101 MHz, CDCl_3_) δ 171.8, 171.2, 165.5, 141.0, 136.4, 135.8, 130.4, 126.4, 94.4, 53.1, 52.3, 49.2, 36.1; ESI-HRMS calcd for C_13_H_14_INNaO_5_ (M + Na)^+^ 413.9809, found 413.9801; [α]^20^_D_ − 22.0° (*c* 0.2, MeOH).

**6** was synthesised from **6e** (27 mg, 0.07 mmol) according to the general ester hydrolysis procedure to give 24 mg (97%) of a white solid: *t*_*R*_ = 8.93 min (HPLC); ^1^H NMR (400 MHz, Acetone-*d*_*6*_) δ 8.25 (t, *J* = 1.7 Hz, 1H), 8.13–8.07 (m, 1H), 7.94–7.89 (m, 2H), 7.30 (t, *J* = 7.8 Hz, 1H), 5.06–4.99 (m, 1H), 3.09–2.93 (m, 2H); ^13^C NMR (101 MHz, Acetone-*d*_*6*_) δ 172.3, 172.2, 165.9, 141.2, 137.3, 137.1, 131.4, 127.6, 94.4, 50.3, 36.4; ESI-HRMS calcd for C_11_H_9_INO_5_ (M − H)^−^ 361.9531, found 361.9531; [α]^20^_D_ + 4.0° (*c* 0.2, MeOH).

### (4-Bromobenzoyl)-*L*-aspartic acid (7)

Dimethyl (4-bromobenzoyl)-*L*-aspartate (**7e**) was synthesised from 4-bromobenzoic acid (133 mg, 0.66 mmol) and **2** (99 mg, 0.50 mmol) according to the general amide coupling procedure. Purification by flash column chromatography (EtOAc:petroleum ether, 1:1) gave 137 mg (80%) of a beige solid: Rf = 0.35 (EtOAc:petroleum ether, 1:1); ^1^H NMR (400 MHz, CDCl_3_) δ 7.71–7.66 (m, 2H), 7.61–7.56 (m, 2H), 7.23–7.17 (m, 1H), 5.07–5.00 (m, 1H), 3.80 (s, 3H), 3.71 (s, 3H), 3.19–2.93 (m, 2H); ^13^C NMR (101 MHz, CDCl_3_) δ 171.9, 171.2, 166.1, 132.7, 132.0, 128.9, 126.8, 53.1, 52.3, 49.1, 36.1; ESI-HRMS calcd for C_13_H_14_BrNNaO_5_ (M + Na)^+^ 365.9948, found 365.9937; [α]^20^_D_ −22.5° (*c* 0.2, MeOH).

**7** was synthesised from **7e** (25 mg, 0.07 mmol) according to the general ester hydrolysis procedure to give 21 mg (93%) of a white solid: *t*_*R*_ = 8.73 min (HPLC); ^1^H NMR (400 MHz, Acetone-*d*_*6*_) δ 10.69 (br s, 2H), 8.09–8.01 (m, 1H), 7.89–7.82 (m, 2H), 7.72–7.65 (m, 2H), 5.07–4.99 (m, 1H), 3.09–2.93 (m, 2H); ^13^C NMR (101 MHz, Acetone-*d*_*6*_) δ 172.4, 172.3, 166.5, 134.3, 132.5, 130.2, 126.5, 50.3, 36.4; ESI-HRMS calcd for C_11_H_9_BrNO_5_ (M − H)^−^ 313.9670, found 313.9674; [α]^20^_D_ + 4.0° (*c* 0.2, MeOH).

### (2-(4-Bromophenyl)acetyl)-*L*-aspartic acid (8)

A dry flask under an argon atmosphere was charged with 4-bromophenylacetic acid (300 mg, 1.4 mmol), **2** (276 mg, 1.4 mmol), DMF (5 mL), *N,N*-diisopropylethylamine (0.85 mL), 1-hydroxybenzotriazole (256 mg, 1.67 mmol), and *N*-(3-dimethylaminopropyl)-*N*′-ethylcarbodiimide hydrochloride (321 mg, 1.67 mmol). The reaction was stirred overnight at rt. After completion, the solvent was evaporated and the residue was extracted with EtOAc (x3). The organic phases were combined, washed with aqueous 1 M HCl, saturated aqueous NaHCO_3_, brine, and dried over Na_2_SO_4_ and concentrated *in vacuo*. The residue was purified by flash chromatography (EtOAc:petroleum ether, 1:1) to give 317 mg (63%) of dimethyl (2-(4-bromophenyl)acetyl)-*L*-aspartate (**8e**) as a white solid: Rf = 0.14 (EtOAc:petroleum ether, 1:1); ^1^H NMR (400 MHz, CDCl_3_) δ 7.50–7.44 (m, 2H), 7.19–7.13 (m, 2H), 6.47 (d, *J* = 7.5 Hz, 1H), 4.86–4.79 (m, 1H), 3.74 (s, 3H), 3.64 (s, 3H), 3.54 (s, 2H), 3.04–2.96 (m, 1H), 2.86–2.78 (m, 1H); ^13^C NMR (101 MHz, CDCl_3_) δ 171.3, 170.9, 170.0, 133.4, 132.0, 131.0, 121.4 52.8, 52.0, 48.7, 42.8, 35.9; ESI-HRMS calcd for C_14_H_17_BrNO_5_ (M + H)^+^ 358.0285, found 358.0295; [α]^20^_D_ − 6.0° (*c* 0.2, MeCN).

**8** was synthesised from **8e** (50 mg, 0.14 mmol) according to the general ester hydrolysis procedure to give 45 mg (98%) of a white solid: *t*_R_ = 8.88 min (HPLC); ^1^H NMR (400 MHz, DMSO-*d*_*6*_) δ 8.44 (d, *J* = 7.9 Hz, 1H), 7.54–7.41 (m, 2H), 7.21 (d, *J* = 8.4 Hz, 2H), 4.55–4.45 (m, 1H), 3.45 (s, 2H), 2.73–2.64 (m, 1H), 2.62–2.53 (m, 1H); ^13^C NMR (101 MHz, DMSO-*d*_*6*_) δ 177.5, 176.8, 174.8, 140.9, 136.5, 136.2, 124.7, 54.0, 46.3, 41.3; ESI-HRMS calcd for C_12_H_12_BrNNaO_5_ (M + Na)^+^ 351.9791, found 351.9775; [α]^20^_D_ + 3.0° (*c* 0.2, MeOH).

### (2-(4′-Fluoro-[1,1′-biphenyl]-4-yl)acetyl)-*L*-aspartic acid (9)

Dimethyl (2-(4′-fluoro-[1,1′-biphenyl]−4-yl)acetyl)-*L*-aspartate (**9e**) was synthesised from **8e** (100 mg, 0.28 mmol) and 4-fluorophenylboronic acid (43 mg, 0.31 mmol) according to the general Suzuki coupling procedure. Purification by flash chromatography (EtOAc:petroleum ether, 1:4) gave 80 mg (77%) of a white solid: Rf = 0.22 (EtOAc:petroleum ether, 1:1); ^1^H NMR (400 MHz, CDCl_3_) δ 7.57–7.49 (m, 4H), 7.34 (d, *J* = 8.4 Hz, 2H), 7.15–7.08 (m, 2H), 6.51 (d, *J* = 7.6 Hz, 1H), 4.89–4.82 (m, 1H), 3.73 (s, 3H), 3.65–2.60 (m, 5H), 3.04–2.96 (m, 1H), 2.88–2.80 (m, 1H); ^13^C NMR (101 MHz, CDCl_3_) δ 171.3, 171.0, 170.5, 160.7 (d, *J* = 292.3 Hz), 139.3, 136.8 (d, *J* = 3.3 Hz), 133.5, 129.8, 128.6 (d, *J* = 8.0 Hz), 127.5, 115.7 (d, *J* = 21.5 Hz), 52.8, 52.0, 48.7, 43.1, 36.0; ESI-HRMS calcd for C_20_H_20_FNNaO_5_ (M + Na)^+^ 396.1218, found 396.1224; [α]^20^_D_ − 5.0° (*c* 0.2, MeCN).

**9** was synthesised from **9e** (73 mg, 0.20 mmol) according to the general ester hydrolysis procedure to give 65 mg (96%) of a white solid: *t*_R_ = 10.25 min (HPLC); ^1^H NMR (400 MHz, Acetone-*d*_*6*_) δ 7.71–7.65 (m, 2H), 7.58–7.54 (m, 2H), 7.42 (d, *J* = 8.1 Hz, 2H), 7.25–7.18 (m, 2H), 4.85–4.79 (m, 1H), 3.65 (s, 2H), 2.90–2.86 (m, 2H); ^13^C NMR (101 MHz, Acetone-*d*_*6*_) δ 176.6, 176.5, 175.6, 167.6 (d, *J* = 244.4 Hz), 143.4, 142.3 (d, *J* = 3.2 Hz), 140.4, 135.0, 133.8 (d, *J* = 8.1 Hz), 131.9, 120.7 (d, *J* = 21.6 Hz), 53.9, 47.1, 40.8; ESI-HRMS calcd for C_18_H_15_FNO_5_ (M + H)^+^ 344.0940, found 344.0949; [α]^20^_D_ + 14.6° (*c* 0.2, MeCN).

### (5-Phenylfuran-2-carbonyl)-*L*-aspartic acid (10)

Dimethyl (5-phenylfuran-2-carbonyl)-*L*-aspartate (**10e**) was synthesised from **4e** (100 mg, 0.30 mmol) and phenylboronic acid (40 mg, 0.33 mmol) according to the general Suzuki coupling procedure. Purification by flash chromatography (EtOAc:petroleum ether, 1:3) gave 67 mg (67%) of a clear oil: Rf = 0.25 (EtOAc:petroleum ether, 1:1); ^1^H NMR (400 MHz, CDCl_3_) δ 7.76–7.72 (m, 2H), 7.46–7.33 (m, 4H), 7.22 (d, *J* = 3.6 Hz, 1H), 6.75 (d, *J* = 3.6 Hz, 1H), 5.10–5.04 (m, 1H), 3.82 (s, 3H), 3.73 (s, 3H), 3.20–3.12 (m 1H), 3.03–2.95 (m, 1H); ^13^C NMR (101 MHz, CDCl_3_) δ 171.6, 171.1, 158.0, 156.0, 146.3, 129.6, 128.9, 128.8, 124.6, 117.2, 107.3, 53.0, 52.1, 48.3, 36.3; ESI-HRMS calcd for C_17_H_18_NO_6_ (M + H)^+^ 332.1129, found 332.1118; [α]^20^_D_ − 7.5° (*c* 0.2, MeCN).

**10** was synthesised from **10e** (50 mg, 0.15 mmol) according to the general ester hydrolysis procedure to give 44 mg (96%) of a white solid: *t*_R_ = 9.38 min (HPLC); ^1^H NMR (400 MHz, Acetone-*d*_*6*_) δ 8.08 (d, *J* = 8.1 Hz, 1H), 7.87–7.80 (m, 2H), 7.49–7.42 (m, 2H), 7.40–7.33 (m, 1H), 7.21 (d, *J* = 3.6 Hz, 1H), 7.01 (d, *J* = 3.6 Hz, 1H), 5.09–5.01 (m, 1H), 3.12–2.97 (m, 2H); ^13^C NMR (101 MHz, Acetone-*d*_*6*_) δ 172.4, 172.3, 158.5, 156.3, 148.0, 130.7, 129.8, 129.5, 125.2, 117.1, 108.2, 49.4, 36.5; ESI-HRMS calcd for C_15_H_13_NNaO_6_ (M + Na)^+^ 326.0635, found 326.0645; [α]^20^_D_ + 3.9° (*c* 0.2, MeCN).

### (5-(*o*-Tolyl)furan-2-carbonyl)-*L*-aspartic acid (11)

Dimethyl (5-(*o*-tolyl)furan-2-carbonyl)-*L*-aspartate (**11e**) was synthesised from **4e** (100 mg, 0.30 mmol) and *o*-tolylboronic acid (45 mg, 0.33 mmol) according to the general Suzuki coupling procedure. Purification by flash chromatography (EtOAc:petroleum ether, 1:3) gave 80 mg (77%) of a clear oil: Rf = 0.29 (EtOAc:petroleum ether, 1:1); ^1^H NMR (400 MHz, CDCl_3_) δ 7.71–7.76 (m, 1H), 7.39 (d, *J* = 8.1 Hz, 1H), 7.32–7.27 (m, 3H), 7.25 (d, *J* = 3.6 Hz, 1H), 6.64 (d, *J* = 3.6 Hz), 5.09–5.03 (m, 1H), 3.80 (s, 3H), 3.71 (s, 3H), 3.19–3.11 (m, 1H), 3.02–3.94 (m, 1H), 2.54 (s, 3H); ^13^C NMR (101 MHz, CDCl_3_) δ 171.5, 171.1, 158.0, 155.8, 146.0, 135.4, 131.4, 129.0, 128.8, 127.8, 126.2, 116.6, 110.6, 52.9, 52.1, 48.2, 36.3, 21.7; ESI-HRMS calcd for C_18_H_19_NNaO_6_ (M + Na)^+^ 368.1105, found 368.1120; [α]^20^_D_ + 23.7° (*c* 0.2, DCM).

**11** was synthesised from **11e** (50 mg, 0.14 mmol) according to the general ester hydrolysis procedure to give 45 mg (98%) of a pale yellow solid: *t*_R_ = 9.85 min (HPLC); ^1^H NMR (400 MHz, Acetone-*d*_*6*_) δ 7.99 (d, *J* = 8.3 Hz, 1H), 7.77–7.73 (m, 1H), 7.34–7.26 (m, 3H), 7.23 (d, *J* = 3.6 Hz, 1H), 6.83 (d, *J* = 3.6 Hz, 1H), 5.08–5.01 (m, 1H), 3.05 (d, *J* = 5.7 Hz, 2H), 2.52 (s, 3H); ^13^C NMR (101 MHz, Acetone-*d*_*6*_) δ 172.5, 172.3, 158.5, 156.1, 147.7, 136.2, 132.2, 130.0, 129.5, 128.4, 127.1, 116.7, 111.6, 49.2, 36.4, 21.9; ESI-HRMS calcd for C_16_H_14_NO_6_ (M + H)^+^ 316.0827, found 316.0816; [α]^20^_D_ + 30.5° (*c* 0.2, MeCN).

### (5-(*m*-Tolyl)furan-2-carbonyl)-*L*-aspartic acid (12)

Dimethyl (5-(*m*-tolyl)furan-2-carbonyl)-*L*-aspartate (**12e**) was synthesised from **4e** (100 mg, 0.30 mmol) and *m*-tolylboronic acid (45 mg, 0.33 mmol) according to the general Suzuki coupling procedure. Purification by flash chromatography (EtOAc:petroleum ether, 1:3) gave 70 mg (68%) of a clear oil: Rf = 0.23 (EtOAc:petroleum ether, 1:1); ^1^H NMR (400 MHz, CDCl_3_) δ 7.56–7.52 (m, 2H), 7.37 (d, *J* = 8.1Hz, 1H), 7.32 (t, *J* = 7.9 Hz, 1H), 7.22 (d, *J* = 3.6 Hz, 1H), 7.17 (d, *J* = 7.6 Hz, 1H), 6.73 (d, *J* = 3.6 Hz, 1H), 5.11–5.04 (m, 1H), 3.82 (s, 3H), 3.73 (s, 3H), 3.20–3.12 (m, 1H), 3.04–2.96 (m, 1H), 2.42 (s, 3H); ^13^C NMR (101 MHz, CDCl_3_) δ 171.6, 171.1, 158.0, 156.2, 146.2, 138.6, 129.7, 129.5, 128.8, 125.2, 121.9, 117.2, 107.2, 53.0, 52.1, 48.3, 36.3, 21.5; ESI-HRMS calcd for C_18_H_19_NaNO_6_ (M + Na)^+^ 368.1105, found 368.1103; [α]^20^_D_ + 19.3° (*c* 0.2, MeCN).

**12** was synthesised from **12e** (50 mg, 0.14 mmol) according to the general ester hydrolysis procedure to give 46 mg (quant.) of a white solid: *t*_R_ = 9.88 min (HPLC); ^1^H NMR (400 MHz, Acetone-*d*_*6*_) δ 8.04 (d, *J* = 8.1 Hz, 1H), 7.68–7.61 (m, 2H), 7.34 (t, *J* = 7.7 Hz, 1H), 7.22–7.17 (m, 2H), 6.99 (d, *J* = 3.6 Hz, 1H), 5.08–5.00 (m, 1H), 3.10–2.96 (m, 2H), 2.37 (s, 3H); ^13^C NMR (101 MHz, Acetone-*d*_*6*_) δ 172.34, 172.27, 156.5, 154.2, 148.0, 139.4, 130.7, 130.3, 129.7, 125.8, 122.5, 117.0, 108.1, 49.4, 36.43, 21.4; ESI-HRMS calcd for C_16_H_14_NO_6_ (M + H)^+^ 316.0827, found 316.0826; [α]^20^_D_ + 7.0° (*c* 0.2, MeCN).

### (5-(*p*-Tolyl)furan-2-carbonyl)-*L*-aspartic acid (13)

Dimethyl (5-(*p*-tolyl)furan-2-carbonyl)-*L*-aspartate (**13e**) was synthesised from **4e** (100 mg, 0.30 mmol) and *p*-tolylboronic acid (45 mg, 0.33 mmol) according to the general Suzuki coupling procedure. Purification by flash chromatography (EtOAc:petroleum ether, 1:3) gave 80 mg (77%) of a clear oil: Rf = 0.26 (EtOAc:petroleum ether, 1:1); ^1^H NMR (400 MHz, CDCl_3_) δ 7.66–7.59 (m, 2H), 7.37 (d, *J* = 8.1 Hz, 1H), 7.23 (d, *J* = 7.9 Hz, 2H), 7.21 (d, *J* = 3.6 Hz, 1H), 6.69 (d, *J* = 3.6 Hz, 1H), 5.10–5.03 (m, 1H), 3.81 (s, 3H), 3.71 (s, 3H), 3.19–3.11 (m, 1H), 3.03–2.95 (m, 1H), 2.39 (s, 3H); ^13^C NMR (101 MHz, CDCl_3_) δ 171.6, 171.1, 158.0, 156.2, 145.9, 138.9, 129.6, 126.9, 124.6, 117.2, 106.6, 52.9, 52.1, 48.3, 36.3, 21.4; ESI-HRMS calcd for C_18_H_19_NaNO_6_ (M + Na)^+^ 368.1105, found 368.1104; [α]^20^_D_ + 15.5° (*c* 0.2, DCM).

**13** was synthesised from **13e** (50 mg, 0.14 mmol) according to the general ester hydrolysis procedure to give 45 mg (98%) of a white solid: *t*_R_ = 9.89 min (HPLC); ^1^H NMR (400 MHz, Acetone-*d*_*6*_) δ 7.74–7.69 (m, 2H), 7.27 (d, *J* = 8.0 Hz, 2H), 7.18 (d, *J* = 3.6 Hz, 1H), 6.94 (d, *J* = 3.6 Hz, 1H), 5.03 (t, *J* = 5.9 Hz, 1H), 3.11–2.96 (m, 2H), 2.35 (s, 3H); ^13^C NMR (101 MHz, Acetone-*d*_*6*_) δ 172.48, 172.45, 158.5, 156.6, 147.7, 139.6, 130.4, 128.0, 125.3, 117.2, 107.5, 49.3, 36.6, 21.3; ESI-HRMS calcd for C_14_H_16_NO_6_ (M + H)^+^ 316.0827, found 316.0821; [α]^20^_D_ + 10.5° (*c* 0.2, MeCN).

### (5-(3-(Hydroxymethyl)phenyl)furan-2-carbonyl)-*L*-aspartic acid (14)

Dimethyl (5-(3-(hydroxymethyl)phenyl)furan-2-carbonyl)-*L*-aspartate (**14e**) was synthesised from **4e** (107 mg, 0.32 mmol) and 3-hydroxymethylphenylboronic acid (54 mg, 0.35 mmol) according to the general Suzuki coupling procedure. Purification by flash chromatography (EtOAc:petroleum ether, 2:1) gave 55 mg (47%) of a clear oil: Rf = 0.29 (EtOAc:petroleum ether, 2:1); ^1^H NMR (400 MHz, CDCl_3_) δ 7.73 (s, 1H), 7.66 (d, *J* = 7.7 Hz, 1H), 7.46–7.33 (m, 3H), 7.21 (d, *J* = 3.6 Hz, 1H), 6.76 (d, *J* = 3.6 Hz, 1H), 5.09–5.03 (m, 1H), 4.76 (s, 2H), 3.81 (s, 3H), 3.73 (s, 3H), 3.19–3.11 (m, 1H), 3.03–2.95 (m, 1H), 1.99 (s, 1H); ^13^C NMR (101 MHz, CDCl_3_) δ 171.6, 171.1, 157.9, 155.8, 146.3, 141.7, 129.8, 129.1, 127.3, 123.8, 123.0, 117.2, 107.5, 65.0, 52.9, 52.1, 48.3, 36.2; ESI-HRMS calcd for C_18_H_20_NO_7_ (M + H)^+^ 362.1234, found 362.1246; [α]^20^_D_ − 8.0° (*c* 0.2, MeCN).

**14** was synthesised from **14e** (50 mg, 0.14 mmol) according to the general ester hydrolysis procedure to give 44 mg (95%) of a white solid: *t*_R_ = 8.04 min (HPLC); ^1^H NMR (400 MHz, Acetone-*d*_*6*_) δ 8.11 (d, *J* = 8.3 Hz, 1H), 7.82 (s, 1H), 7.70 (d, *J* = 7.5 Hz, 1H), 7.45–7.33 (m, 2H), 7.21 (d, *J* = 3.6 Hz, 1H), 7.00 (d, *J* = 3.6 Hz, 1H), 5.09–5.01 (m, 1H), 4.68 (s, 2H), 3.10–2.97 (m, 2H), 1.31–1.25 (m, 1H); ^13^C NMR (101 MHz, Acetone-*d*_*6*_) δ 172.5, 172.4, 158.6, 156.6, 147.9, 144.2, 130.6, 129.7, 127.8, 123.8, 123.4, 117.2, 108.2, 64.5, 49.4, 36.5; ESI-HRMS calcd for C_16_H_14_NO_7_ (M + H)^+^ 332.0776, found 332.0760; [α]^20^_D_ + 14.5° (*c* 0.2, MeCN).

### (5-(4-Hydroxyphenyl)furan-2-carbonyl)-*L*-aspartic acid (15)

Dimethyl (5-(4-hydroxyphenyl)furan-2-carbonyl)-*L*-aspartate (**15e**) was synthesised from **4e** (100 mg, 0.30 mmol) and 4-hydroxyphenylboronic acid (45 mg, 0.33 mmol) according to the general Suzuki coupling procedure. Purification by flash chromatography (EtOAc:petroleum ether, 1:2) gave 30 mg (29%) of a clear oil: Rf = 0.23 (EtOAc:petroleum ether, 1:1); ^1^H NMR (400 MHz, CDCl_3_) δ 7.64–7.56 (m, 2H), 7.37 (d, *J* = 8.1 Hz, 1H), 7.21 (d, *J* = 3.6 Hz, 1H), 6.94–6.87 (m, 2H), 6.59 (d, *J* = 3.6 Hz, 1H), 5.79 (s, 1H), 5.10–5.04 (m, 1H), 3.82 (s, 3H), 3.73 (s, 3H), 3.20–3.12 (m, 1H), 3.03–2.95 (m, 1H); ^13^C NMR (101 MHz, CDCl_3_) δ 171.7, 171.2, 158.2, 156.6, 156.3, 145.5, 126.4, 122.5, 117.5, 115.9, 105.8, 53.0, 52.1, 48.3, 36.3; ESI-HRMS calcd for C_17_H_18_NO_7_ (M + H)^+^ 348.1078, found 348.1083; [α]^20^_D_ − 3.5° (*c* 0.2, MeCN).

**15** was synthesised from **15e** (27 mg, 0.08 mmol) according to the general ester hydrolysis procedure to give 24 mg (97%) of a pale brown solid: *t*_R_ = 8.15 min (HPLC); ^1^H NMR (400 MHz, Acetone-*d*_*6*_) δ 7.99 (d, *J* = 8.2 Hz, 1H), 7.71–7.64 (m, 2H), 7.16 (d, *J* = 3.6 Hz, 1H), 6.94–6.88 (m, 2H), 6.80 (d, *J* = 3.6 Hz, 1H), 5.07–4.99 (m, 1H), 3.09–2.97 (m, 2H); ^13^C NMR (101 MHz, Acetone-*d*_*6*_) δ 172.41, 172.36, 159.0, 158.6, 156.9, 147.1, 127.0, 122.5, 117.2, 116.7, 106.1, 49.3, 36.5; ESI-HRMS calcd for C_15_H_12_NO_7_ (M + H)^+^ 318.0619, found 318.0615; [α]^20^_D_ + 5.4° (*c* 0.2, MeCN).

### (5-(2-Methoxyphenyl)furan-2-carbonyl)-*L*-aspartic acid (16)

Dimethyl (5-(2-methoxyphenyl)furan-2-carbonyl)-*L*-aspartate (**16e**) was synthesised from **4e** (100 mg, 0.30 mmol) and *o*-methoxyphenylboronic acid (40 mg, 0.33 mmol) according to the general Suzuki coupling procedure. Purification by flash chromatography (EtOAc:petroleum ether, 1:3) gave 61 mg (56%) of a clear oil: Rf = 0.26 (EtOAc:petroleum ether, 1:1); ^1^H NMR (400 MHz, CDCl_3_) δ 7.89 (dd, *J* = 7.8, 1.7 Hz, 1H), 7.39 (d, *J* = 8.1 Hz, 1H), 7.33 (ddd, *J* = 8.4, 7.4, 1.7 Hz, 1H), 7.23 (d, *J* = 3.6 Hz, 1H), 7.07 (td, *J* = 7.7, 1.0 Hz, 1H), 7.03 (d, *J* = 3.6 Hz, 1H), 6.99 (d, *J* = 8.3 Hz, 1H), 5.12–5.01 (m, 1H), 3.96 (s, 3H), 3.81 (s, 3H), 3.73 (s, 3H), 3.20–3.12 (m, 1H), 3.03–2.95 (m, 1H); ^13^C NMR (101 MHz, CDCl_3_) δ 171.6, 171.2, 158.1, 156.3, 152.5, 145.2, 129.6, 126.6, 120.9, 118.6, 117.2, 112.1, 111.2, 55.5, 52.9, 52.1, 48.3, 36.3; ESI-HRMS calcd for C_18_H_20_NO_7_ (M + H)^+^ 362.1234, found 362.1249; [α]^20^_D_ − 5.9° (*c* 0.2, MeCN).

**16** was synthesised from **16e** (49 mg, 0.13 mmol) according to the general ester hydrolysis procedure to give 40 mg (89%) of a white solid: *t*_R_ = 9.59 min (HPLC); ^1^H NMR (400 MHz, Acetone-*d*_*6*_) δ 8.07 (d, *J* = 8.3 Hz, 1H), 7.93 (dd, *J* = 7.8, 1.7 Hz, 1H), 7.35 (ddd, *J* = 8.4, 7.4, 1.7 Hz, 1H), 7.19 (d, *J* = 3.6 Hz, 1H), 7.14 (d, *J* = 8.4, 0.8 Hz, 1H), 7.08 (d, *J* = 3.6 Hz, 1H), 7.04 (td, *J* = 7.7, 1.1 Hz, 1H), 5.08–5.02 (m, 1H), 3.99 (s, 3H), 3.10–2.98 (m, 2H); ^13^C NMR (101 MHz, Acetone-*d*_*6*_) δ 172.5, 172.4, 158.6, 157.3, 152.9, 146.9, 130.5, 127.0, 121.5, 119.4, 117.1, 112.8, 112.5, 55.9, 49.4, 36.5; ESI-HRMS calcd for C_16_H_15_NNaO_7_ (M + Na)^+^ 356.0741, found 356.0743; [α]^20^_D_ + 3.0° (*c* 0.2, MeCN).

### (5-(4-Methoxyphenyl)furan-2-carbonyl)-*L*-aspartic acid (17)

Dimethyl (5-(4-methoxyphenyl)furan-2-carbonyl)-*L*-aspartate (**17e**) was synthesised from **4e** (100 mg, 0.30 mmol) and *p*-methoxyphenylboronic acid (40 mg, 0.33 mmol) according to the general Suzuki coupling procedure. Purification by flash chromatography (EtOAc:petroleum ether, 1:3) gave 89 mg (82%) of a clear oil: Rf = 0.20 (EtOAc:petroleum ether, 1:1); ^1^H NMR (400 MHz, CDCl_3_) δ 7.69–7.64 (m, 2H), 7.35 (d, *J* = 8.0 Hz, 1H), 7.20 (d, *J* = 3.60 Hz, 1H), 6.99–6.92 (m, 2H), 6.61 (d, *J* = 3.6 Hz, 1H), 5.09–5.03 (m, 1H), 3.86 (s, 3H), 3.81 (s, 3H), 3.71 (s, 3H), 3.19–3.11 (m, 1H), 3.02–2.94 (m, 1H); ^13^C NMR (101 MHz, CDCl_3_) δ 171.6, 171.2, 160.2, 158.0, 156.1, 145.7, 126.2, 122.5, 117.3, 114.4, 105.8, 55.4, 52.9, 52.1, 48.2, 36.3; ESI-HRMS calcd for C_18_H_20_NO_7_ (M + H)^+^ 362.1234, found 362.1249, [α]^20^_D_ − 6.0° (*c* 0.2, MeCN).

**17** was synthesised from **17e** (75 mg, 0.21 mmol) according to the general ester hydrolysis procedure to give 66 mg (96%) of a white solid: *t*_R_ = 9.48 min (HPLC); ^1^H NMR (400 MHz, Acetone-*d*_*6*_) δ 8.05 (d, *J* = 8.2 Hz, 1H), 7.75 (d, *J* = 8.8 Hz, 2H), 7.19 (d, *J* = 3.6 Hz, 1H), 7.00 (d, *J* = 8.8 Hz, 2H), 6.84 (d, *J* = 3.6 Hz, 1H), 5.09–5.00 (m, 1H), 3.83 (s, 3H), 3.11–2.98 (m, 2H); ^13^C NMR (101 MHz, Acetone-*d*_*6*_) δ 172.5, 172.4, 161.2, 158.7, 156.7, 147.3, 126.9, 123.5, 117.4, 115.3, 106.6, 55.8, 49.4, 36.5; ESI-HRMS calcd for C_16_H_15_NNaO_7_ (M + Na)^+^ 356.0741, found 356.0755; [α]^20^_D_ + 2.5° (*c* 0.2, MeCN).

### (5-(4-(Trifluoromethyl)phenyl)furan-2-carbonyl)-*L*-aspartic acid (18)

Dimethyl (5-(4-(trifluoromethyl)phenyl)furan-2-carbonyl)-*L*-aspartate (**18e**) was synthesised from **4e** (100 mg, 0.30 mmol) and 4-trifluoromethylphenylboronic acid (63 mg, 0.33 mmol) according to the general Suzuki coupling procedure. Purification by flash chromatography (EtOAc:petroleum ether, 1:3) gave 67 mg (56%) of a clear oil: Rf = 0.59 (EtOAc:petroleum ether, 2:1); ^1^H NMR (400 MHz, CDCl_3_) δ 7.83 (d, *J* = 8.6 Hz, 2H), 7.68 (d, *J* = 8.6 Hz, 2H), 7.44 (d, *J* = 8.1 Hz, 1H), 7.25 (d, *J* = 3.6 Hz, 1H), 6.86 (d, *J* = 3.6 Hz, 1H), 5.09–5.03 (m, 1H), 3.82 (s, 3H), 3.74 (s, 3H), 3.21–3.13 (m, 1H), 3.03–2.95 (m, 1H); ^13^C NMR (101 MHz, CDCl_3_) δ 171.6, 171.0, 157.7, 154.2, 147.2, 132.7, 130.5 (q, *J* = 32.7 Hz), 126.0 (q, *J* = 3.8 Hz), 125.3, 124.7, 117.1, 109.1, 53.0, 52.2, 48.3, 36.2; ESI-HRMS calcd for C_18_H_16_F_3_NaNO_6_ (M + Na)^+^ 422.0822, found 422.0832; [α]^20^_D_ − 5.0° (*c* 0.2, MeCN).

**18** was synthesised from **18e** (50 mg, 0.13 mmol) according to the general ester hydrolysis procedure to give 46 mg (99%) of a white solid: *t*_R_ = 10.73 min (HPLC); ^1^H NMR (400 MHz, Acetone-*d*_*6*_) δ 8.19 (d, *J* = 8.2 Hz, 1H), 8.02 (t, *J* = 9.7 Hz, 2H), 7.78 (d, *J* = 8.3 Hz, 2H), 7.26 (d, *J* = 3.6 Hz, 1H), 7.21 (d, *J* = 3.6 Hz, 1H), 5.10–5.01 (m, 1H), 3.08–3.00 (m, 2H); ^13^C NMR (101 MHz, Acetone-*d*_*6*_) δ 172.4, 172.3, 158.4, 154.6, 148.9, 134.2, 130.4 (q, *J* = 32.3 Hz), 126.8 (q, *J* = 3.8 Hz), 125.7, 117.2, 110.5, 49.5, 36.5; ESI-HRMS calcd for C_16_H_11_F_3_NO_6_ (M + H)^+^ 370.0544, found 370.0528; [α]^20^_D_ + 3.5° (*c* 0.2, MeCN).

### (5-(4-Cyanophenyl)furan-2-carbonyl)-*L*-aspartic acid (19)

Dimethyl (5-(4-cyanophenyl)furan-2-carbonyl)-*L*-aspartate (**19e**) was synthesised from **4e** (100 mg, 0.30 mmol) and 4-cyanophenylboronic acid (48 mg, 0.33 mmol) according to the general Suzuki coupling procedure. Purification by flash chromatography (EtOAc:petroleum ether, 1:3) gave 70 mg (66%) of a clear oil: Rf = 0.21 (EtOAc:petroleum ether, 1:1); ^1^H NMR (400 MHz, CDCl_3_) δ 7.85–7.79 (m, 2H), 7.75–7.69 (m, 2H), 7.44 (d, *J* = 8.0 Hz, 1H), 7.25 (d, *J* = 3.7 Hz, 1H), 6.90 (d, *J* = 3.7 Hz, 1H), 5.08–5.02 (m, 1H), 3.82 (s, 3H), 3.74 (s, 3H), 3.21–3.13 (m, 1H), 3.03–2.95 (m, 1H); ^13^C NMR (101 MHz, CDCl_3_) δ 171.6, 170.9, 157.5, 153.6, 147.6, 133.3, 132.8, 124.9, 118.5, 117.1, 112.0, 110.0, 53.0, 52.2, 48.3, 36.1; ESI-HRMS calcd for C_18_H_16_N_2_NaO_6_ (M + Na)^+^ 379.0901, found 379.0892; [α]_D_^20^ – 9.5° (*c* 0.2, MeCN).

**19** was synthesised from **19e** (57 mg, 0.16 mmol) according to the general ester hydrolysis procedure to give 50 mg (95%) of a pale yellow solid: *t*_R_ = 9.16 min (HPLC); ^1^H NMR (400 MHz, Acetone-*d*_*6*_) δ 8.18 (d, *J* = 8.2 Hz, 1H), 8.06–8.12 (m, 2H), 7.89–7.85 (m, 2H), 7.27 (d, *J* = 3.6 Hz, 1H), 7.25 (d, *J* = 3.6 Hz, 1H), 5.06–5.00 (m, 1H), 3.07–2.98 (m, 2H); ^13^C NMR (101 MHz, Acetone-*d*_*6*_) δ 172.3, 172.2, 158.2, 154.2, 145.9, 134.5, 133.7, 125.7, 119.1, 117.2, 112.4, 111.2, 49.5, 36.4; ESI-HRMS calcd for C_16_H_11_N_2_O_6_ (M + H)^+^ 327.0623, found 327.0628; [α]_D_^20^ + 15.8° (*c* 0.2, MeCN).

### (5-(4-(Trifluoromethoxy)phenyl)furan-2-carbonyl)-*L*-aspartic acid (20)

Dimethyl (5-(4-(trifluoromethoxy)phenyl)furan-2-carbonyl)-*L*-aspartate (**20e**) was synthesised from **4e** (100 mg, 0.30 mmol) and 4-trifluoromethoxyphenylboronic acid (68 mg, 0.33 mmol) according to the general Suzuki coupling procedure. Purification by flash chromatography (EtOAc:petroleum ether, 1:3) gave 54 mg (43%) of a clear oil: Rf = 0.32 (EtOAc:petroleum ether, 1:1); ^1^H NMR (400 MHz, CDCl_3_) δ 7.78–7.73 (m, 2H), 7.40 (d, *J* = 8.0 Hz, 1H), 7.28 (d, *J* = 8.5 Hz, 2H), 7.23 (d, *J* = 3.4 Hz, 1H), 6.75 (d, *J* = 3.6 Hz, 1H), 5.09–5.03 (m, 1H), 3.82 (s, 3H), 3.73 (s, 3H), 3.20–3.12 (m, 1H), 3.02–2.94 (m, 1H); ^13^C NMR (101 MHz, CDCl_3_) δ 171.6, 171.1, 157.8, 154.6, 149.4, 146.7, 135.5, 128.3, 126.1, 121.4, 117.2, 107.9, 53.0, 52.1, 48.3, 36.2; ESI-HRMS calcd for C_18_H_16_F_3_NNaO_7_ (M + Na)^+^ 438.0771, found 438.0782; [α]^20^_D_ − 8.0° (*c* 0.2, MeCN).

**20** was synthesised from **20e** (46 mg, 0.11 mmol) according to the general ester hydrolysis procedure to give 41 mg (95%) of a white solid: *t*_R_ = 10.88 min (HPLC); ^1^H NMR (400 MHz, Acetone-*d*_*6*_) δ 8.00–7.92 (m, 2H), 7.46–7.38 (m, 2H), 7.22 (d, *J* = 3.6 Hz, 1H), 7.08 (d, *J* = 3.6 Hz, 1H), 5.03 (t, *J* = 5.9 Hz, 1H), 3.07–2.99 (m, 2H); ^13^C NMR (101 MHz, Acetone-*d*_*6*_) δ 172.39, 172.36, 158.4, 154.8, 149.8, 148.5, 129.9, 127.1, 122.4, 117.2, 109.1, 49.4, 36.5; ESI-HRMS calcd for C_16_H_11_F_3_NO_7_ (M + H)^+^ 386.0493, found 386.050; [α]^20^_D_ + 6.5° (*c* 0.2, MeCN).

### (5-(4-Ethoxyphenyl)furan-2-carbonyl)-*L*-aspartic acid (21)

Dimethyl (5-(4-ethoxyphenyl)furan-2-carbonyl)-*L*-aspartate (**21e**) was synthesised from **15e** (61 mg, 0.18 mmol) and ethyl iodide (50 µL, 0.62 mmol) according to the general alkylation procedure. After one day of stirring, additional ethyl iodide (50 µL, 0.62 mmol) was added. Purification by flash column chromatography (EtOAc:petroleum ether, 1:1) gave 55 mg (95%) of a pale yellow solid: Rf = 0.22 (EtOAc:petroleum ether, 1:1); ^1^H NMR (400 MHz, Acetone-*d*_*6*_) δ 8.09–8.02 (m, 1H), 7.78–7.73 (m, 2H), 7.16 (d, *J* = 3.6 Hz, 1H), 7.03–6.98 (m, 2H), 6.85 (d, *J* = 3.6 Hz, 1H), 5.07–4.99 (m, 1H), 4.10 (q, *J* = 7.0 Hz, 2H), 3.71 (s, 3H), 3.67 (s, 3H), 3.09–2.91 (m, 3H), 1.38 (t, *J* = 7.0 Hz, 3H); ^13^C NMR (101 MHz, Acetone-*d*_*6*_) δ 172.0, 171.7, 160.6, 158.4, 156.7, 147.3, 126.9, 123.4, 117.3, 115.8, 106.5, 64.3, 52.8, 52.1, 49.6, 36.7, 15.0; ESI-HRMS calcd for C_19_H_21_NNaO_7_ (M + Na)^+^ 398.1210, found 398.1223; [α]^20^_D_ − 4.5° (*c* 0.2, MeOH).

**21** was synthesised from **21e** (44 mg, 0.12 mmol) according to the general ester hydrolysis procedure to give 37 mg (89%) of a pale yellow foam: *t*_*R*_ = 10.01 min (HPLC); ^1^H NMR (400 MHz, Acetone-*d*_*6*_) δ 10.93 (br s, 2H), 8.05–7.97 (m, 1H), 7.80–7.71 (m, 2H), 7.16 (d, *J* = 3.6 Hz, 1H), 7.04–6.97 (m, 2H), 6.85 (d, *J* = 3.6 Hz, 1H), 5.08–5.00 (m, 1H), 4.10 (q, *J* = 7.0 Hz, 2H), 3.12–2.96 (m, 2H), 1.38 (t, *J* = 7.0 Hz, 3H); ^13^C NMR (101 MHz, Acetone-*d*_*6*_) δ 172.42, 172.38, 160.6, 158.6, 156.6, 147.4, 126.9, 123.4, 117.2, 115.8, 106.5, 64.3, 49.4, 36.5, 15.1; ESI-HRMS calcd for C_17_H_16_NO_7_ (M − H)^−^ 346.0932, found 346.0945; [α]^20^_D_ + 6.5° (*c* 0.2, MeOH).

### (5-(4-Isopropoxyphenyl)furan-2-carbonyl)-*L*-aspartic acid (22)

Dimethyl (5-(4-isopropoxyphenyl)furan-2-carbonyl)-*L*-aspartate (**22e**) was synthesised from **15e** (60 mg, 0.17 mmol) and 2-bromopropane (50 µL, 0.53 mmol) according to the general alkylation procedure. In addition, KI (6 mg, 0.03 mmol) was added to promote the reaction. After one day of stirring, additional 2-bromopropane (50 µL, 0.53 mmol) was added. Purification by flash column chromatography (EtOAc:petroleum ether, 1:1) gave 11 mg (16%) of a pale yellow solid: Rf = 0.25 (EtOAc:petroleum ether, 1:1); ^1^H NMR (400 MHz, Acetone-*d*_*6*_) δ 8.08–8.00 (m, 1H), 7.77–7.71 (m, 2H), 7.16 (d, *J* = 3.6 Hz, 1H), 7.03–6.96 (m, 2H), 6.85 (d, *J* = 3.6 Hz, 1H), 5.08–4.98 (m, 1H), 4.74–4.64 (m, 1H), 3.71 (s, 3H), 3.67 (s, 3H), 3.09–2.91 (m, 2H), 1.32 (d, *J* = 6.0 Hz, 6H); ^13^C NMR (101 MHz, Acetone-*d*_*6*_) δ 172.0, 171.7, 159.5, 158.5, 156.7, 147.2, 126.9, 123.2, 117.3, 116.9, 106.5, 70.6, 52.8, 52.1, 49.6, 36.7, 22.3; ESI-HRMS calcd for C_20_H_23_NNaO_7_ (M + Na)^+^ 412.1367, found 412.1387.

**22** was synthesised from **22e** (11 mg, 0.03 mmol) according to the general ester hydrolysis procedure to give 9 mg (86%) of a pale yellow solid: *t*_*R*_ = 10.50 min (HPLC); ^1^H NMR (400 MHz, Acetone-*d*_*6*_) δ 11.19 (br s, 2H), 8.02–7.97 (m, 1H), 7.78–7.71 (m, 2H), 7.16 (d, *J* = 3.6 Hz, 1H), 7.03–6.96 (m, 2H), 6.85 (d, *J* = 3.6 Hz, 1H), 5.09–4.98 (m, 1H), 4.74–4.63 (m, 1H), 3.10–2.95 (m, 2H), 1.32 (d, *J* = 6.0 Hz, 6H); ^13^C NMR (101 MHz, Acetone-*d*_*6*_) δ 172.41, 172.38, 159.5, 158.6, 156.7, 147.4, 126.9, 123.3, 117.2, 116.9, 106.5, 70.6, 49.4, 36.5, 22.3; ESI-HRMS calcd for C_18_H_18_NO_7_ (M − H)^−^ 360.1089, found 360.1103; [α]^20^_D_ + 57.0° (*c* 0.2, MeOH).

### (5-(4-((3-Methyloxetan-3-yl)methoxy)phenyl)furan-2-carbonyl)-*L*-aspartic acid (23)

Dimethyl (5-(4-((3-methyloxetan-3-yl)methoxy)phenyl)furan-2-carbonyl)-*L*-aspartate (**23e**) was synthesised from **15e** (55 mg, 0.35 mmol) and (3-methyloxetan-3-yl)methyl 4-methylbenzenesulfonate (89 mg, 0.53 mmol) according to the general alkylation procedure. In addition, KI (10 mg, 0.06 mmol) was added to promote the reaction. Purification by flash column chromatography (EtOAc:petroleum ether, 1:1) gave 38 mg (55%) of a yellow solid: Rf = 0.17 (EtOAc:petroleum ether, 1:1); ^1^H NMR (400 MHz, Acetone-*d*_*6*_) δ 8.10–8.03 (m, 1H), 7.82–7.75 (m, 2H), 7.17 (d, *J* = 3.6 Hz, 1H), 7.12–7.06 (m, 2H), 6.88 (d, *J* = 3.6 Hz, 1H), 5.09–4.98 (m, 1H), 4.55 (d, *J* = 5.8 Hz, 2H), 4.35 (d, *J* = 5.8 Hz, 2H), 4.16 (s, 2H), 3.71 (s, 3H), 3.67 (s, 3H), 3.09–2.89 (m, 2H), 1.43 (s, 3H); ^13^C NMR (101 MHz, Acetone-*d*_*6*_) δ 171.9, 171.7, 160.8, 158.4, 156.6, 147.4, 126.9, 123.8, 117.3, 116.0, 106.7, 79.7, 73.9, 52.8, 52.1, 49.6, 40.4, 36.7, 21.5; ESI-HRMS calcd for C_22_H_26_NO_8_ (M + H)^+^ 432.1653, found 432.1668; [α]^20^_D_ + 0.5° (*c* 0.2, MeOH).

**23** was synthesised from **23e** (30 mg, 0.07 mmol) according to the general ester hydrolysis procedure to give 26 mg (92%) of a pale yellow foam: *t*_*R*_ = 9.55 min (HPLC); ^1^H NMR (400 MHz, Acetone-*d*_*6*_) δ 10.93 (br s, 2H), 8.06–7.98 (m, 1H), 7.83–7.75 (m, 2H), 7.17 (d, *J* = 3.6 Hz, 1H), 7.13–7.05 (m, 2H), 6.88 (d, *J* = 3.6 Hz, 1H), 5.07–5.00 (m, 1H), 4.56 (d, *J* = 5.8 Hz, 2H), 4.35 (d, *J* = 5.8 Hz, 2H), 4.15 (s, 2H), 3.12–2.97 (m, 2H), 1.43 (s, 3H); ^13^C NMR (101 MHz, Acetone-*d*_*6*_) δ 172.41, 172.38, 160.8, 158.6, 156.5, 147.5, 126.9, 123.8, 117.2, 116.0, 106.7, 79.7, 73.8, 49.4, 40.4, 36.5, 21.5; ESI-HRMS calcd for C_20_H_20_NO_8_ (M − H)^−^ 402.1194, found 402.1197; [α]^20^_D_ + 10.0° (*c* 0.2, MeOH).

### (5-(4-(3-(Methylsulfonyl)propoxy)phenyl)furan-2-carbonyl)-*L*-aspartic acid (24)

Dimethyl (5-(4-(3-(methylsulfonyl)propoxy)phenyl)furan-2-carbonyl)-*L*-aspartate (**24e**) was synthesised from **15e** (40 mg, 0.12 mmol) and 3-(methylsulfonyl)propyl 4-methylbenzenesulfonate (69 mg, 0.24 mmol) according to the general alkylation procedure. In addition, KI (16 mg, 0.09 mmol) was added to promote the reaction. Purification by flash column chromatography (EtOAc:petroleum ether, 2:1) gave 35 mg (65%) of a white solid: Rf = 0.11 (EtOAc:petroleum ether, 2:1); ^1^H NMR (400 MHz, Acetone-*d*_*6*_) δ 8.09–8.01 (m, 1H), 7.80–7.74 (m, 2H), 7.16 (d, *J* = 3.6 Hz, 1H), 7.08–7.02 (m, 2H), 6.87 (d, *J* = 3.6 Hz, 1H), 5.07–4.99 (m, 1H), 4.23 (t, *J* = 6.1 Hz, 2H), 3.71 (s, 3H), 3.67 (s, 3H), 3.35–3.27 (m, 2H), 3.07–2.92 (m, 2H), 2.99 (s, 3H), 2.35–2.25 (m, 2H); ^13^C NMR (101 MHz, Acetone-*d*_*6*_) δ 171.9, 171.7, 160.2, 158.4, 156.5, 147.4, 126.9, 123.8, 117.3, 115.9, 106.7, 66.9, 52.8, 52.1, 51.9, 49.6, 40.8, 36.7, 23.5; ESI-HRMS calcd for C_21_H_26_NO_9_S (M + H)^+^ 468.1323, found 468.1339; [α]^20^_D_ − 9.0° (*c* 0.2, MeOH).

**24** was synthesised from **24e** (25 mg, 0.05 mmol) according to the general ester hydrolysis procedure to give 24 mg (99%) of a white solid: *t*_*R*_ = 8.76 min (HPLC); ^1^H NMR (400 MHz, DMSO-*d*_*6*_) δ 12.66 (br s, 2H), 8.72–8.64 (m, 1H), 7.90–7.81 (m, 2H), 7.18 (d, *J* = 3.6 Hz, 1H), 7.05 (d, *J* = 8.9 Hz, 2H), 6.96 (d, *J* = 3.6 Hz, 1H), 4.82–4.73 (m, 1 H), 4.15 (t, *J* = 6.2 Hz, 2H), 3.38–3.25 (m, 2H), 3.03 (s, 3H), 2.94–2.68 (m, 2H), 2.24–2.11 (m, 2H); ^13^C NMR (101 MHz, DMSO-*d*_*6*_) δ 172.3, 171.8, 158.6, 157.4, 154.8, 145.9 126.0, 122.3, 116.3, 114.9, 106.0, 65.8, 50.5, 48.5, 40.2, 35.8, 21.9; ESI-HRMS calcd for C_19_H_20_NO_9_S (M − H)^−^ 438.0864, found 438.0871; [α]^20^_D_ + 15.0° (*c* 0.2, MeOH).

### (4′-Fluoro-[1,1′-biphenyl]-3-carbonyl)-*L*-aspartic acid (25)

Dimethyl (4′-fluoro-[1,1′-biphenyl]-3-carbonyl)-*L*-aspartate (**25e**) was synthesised from (4-fluorophenyl)boronic acid (36 mg, 0.26 mmol) and **6e** (79 mg, 0.20 mmol) according to the general Suzuki coupling procedure. Purification by flash column chromatography (EtOAc:petroleum ether, 1:1) gave 25 mg (35%) of a pale yellow sticky oil: Rf = 0.36 (EtOAc:petroleum ether, 1:1); ^1^H NMR (400 MHz, Acetone-*d*_*6*_) δ 8.22–8.15 (m, 1H), 8.15–8.12 (m, 1H), 7.91–7.86 (m, 1H), 7.84–7.80 (m, 1H), 7.77–7.70 (m, 2H), 7.57 (t, *J* = 7.8 Hz, 1H), 7.30–7.22 (m, 2H), 5.10–5.01 (m, 1H), 3.71 (s, 3H), 3.66 (s, 3H), 3.08–2.90 (m, 2H); ^13^C NMR (101 MHz, Acetone-*d*_*6*_) δ 172.0, 171.6, 167.2, 164.9, 141.1, 137.5 (d, *J* = 3.4 Hz), 135.8, 130.8, 130.0, 129.9 (d, *J* = 8.2 Hz), 127.2, 126.6, 116.6 (d, *J* = 21.6 Hz), 52.7, 52.1, 50.5, 36.7; ESI-HRMS calcd for C_19_H_18_FNNaO_5_ (M + Na)^+^ 382.1061, found 382.1053.

**25** was synthesised from **25e** (25 mg, 0.07 mmol) according to the general ester hydrolysis procedure to give 20 mg (87%) of a white foam: *t*_*R*_ = 10.03 min (HPLC); ^1^H NMR (400 MHz, Acetone-*d*_*6*_) δ 8.18–8.10 (m, 2H), 7.89 (dd, *J* = 7.7, 1.1 Hz, 1H), 7.85–7.79 (m, 1H), 7.78–7.71 (m, 2H), 7.61–7.54 (m, 1H), 7.31–7.20 (m, 1H), 5.11–5.02 (m, 1H), 3.10–2.94 (m, 2H); ^13^C NMR (101 MHz, Acetone-*d*_*6*_) δ 172.5, 172.3, 167.3, 163.6 (d, *J* = 245.2 Hz), 141.1, 137.5 (d, *J* = 3.1 Hz), 135.9, 130.8, 130.0, 129.9 (d, *J* = 8.2 Hz), 127.2, 126.6, 116.6 (d, *J* = 21.7 Hz), 50.3, 36.5; ESI-HRMS calcd for C_17_H_13_FNO_5_ (M − H)^−^ 330.0783, found 330.0796; [α]^20^_D_ + 9.0° (*c* 0.2, MeOH).

### (4′-Fluoro-[1,1′-biphenyl]-4-carbonyl)-*L*-aspartic acid (26)

Dimethyl (4′-fluoro-[1,1′-biphenyl]-4-carbonyl)-*L*-aspartate (**26e**) was synthesised from (4-fluorophenyl)boronic acid (37 mg, 0.26 mmol) and **7e** (81 mg, 0.34 mmol) according to the general Suzuki coupling procedure. Purification by flash column chromatography (EtOAc:petroleum ether, 1:1) gave 71 mg (84%) of a white solid: Rf = 0.31 (EtOAc:petroleum ether, 1:1); ^1^H NMR (400 MHz, Acetone-*d*_*6*_) δ 8.10–8.04 (m, 1H), 8.00–7.95 (m, 2H), 7.80–7.72 (m, 4H), 7.30–7.22 (m, 2H), 5.09–5.02 (m, 1H), 3.72 (s, 3H), 3.67 (s, 3H), 3.08–2.93 (m, 2H); ^13^C NMR (101 MHz, Acetone-*d*_*6*_) δ 172.1, 171.7, 166.9, 163.8 (d, *J* = 245.6 Hz), 143.9, 137.2 (d, *J* = 3.1 Hz), 133.9, 129.9 (d, *J* = 8.2 Hz), 128.9, 127.7, 116.6 (d, *J* = 21.7 Hz), 52.7, 52.1, 50.4, 36.7; ESI-HRMS calcd for C_19_H_18_FNNaO_5_ (M+Na)^+^ 382.1061, found 382.1056; [α]^20^_D_ − 27.0° (*c* 0.2, MeOH).

**26** was synthesised from **26e** (56 mg, 0.15 mmol) according to the general ester hydrolysis procedure to give 48 mg (94%) of a white solid: *t*_*R*_ = 10.02 min (HPLC); ^1^H NMR (400 MHz, DMSO-*d*_*6*_) δ 12.58 (br s, 2H), 8.83–8.75 (m, 1H), 8.02–7.92 (m, 2H), 7.85–7.75 (m, 4H), 7.39–7.28 (m, 2H), 4.83–4.74 (m, 1H), 2.93–2.69 (m, 2H); ^13^C NMR (101 MHz, DMSO-*d*_*6*_) δ 172.5, 171.7, 165.6, 161.0, 141.8, 135.6 (d, *J* = 3.1 Hz), 132.6, 128.9 (d, *J* = 8.3 Hz), 128.0, 126.4, 115.8 (d, *J* = 21.4 Hz), 49.4, 35.8; ESI-HRMS calcd for C_17_H_13_FNO_5_ (M − H)^−^ 330.0783, found 330.0768; [α]^20^_D_ + 1.0° (*c* 0.2, MeOH).

### (6-(4-Fluorophenyl)picolinoyl)-*L*-aspartic acid (27)

Dimethyl (6-(4-fluorophenyl)picolinoyl)-*L*-aspartate (**27e**) was synthesised from (4-fluorophenyl)boronic acid (40 mg, 0.28 mmol) and dimethyl (6-bromopicolinoyl)-*L*-aspartate (88 mg, 0.26 mmol) according to the general Suzuki coupling procedure. Purification by flash column chromatography (EtOAc:petroleum ether, 1:1) gave 81 mg (88%) of a pale yellow sticky oil: Rf = 0.62 (EtOAc); ^1^H NMR (400 MHz, Acetone-*d*_*6*_) δ 9.18–9.11 (m, 1H), 8.29–8.23 (m, 2H), 8.18–8.13 (m, 1H), 8.12–8.07 (m, 2H), 7.35–7.26 (m, 2H), 5.13–5.04 (m, 1H), 3.74 (s, 3H), 3.69 (s, 3H), 3.11–3.04 (m, 2H); ^13^C NMR (101 MHz, Acetone-*d*_*6*_) δ 172.0, 171.9, 164.7 (d, *J* = 247.5 Hz), 164.5, 155.7, 150.5, 139.8, 135.5 (d, *J* = 3.1 Hz), 130.0 (d, *J* = 8.5 Hz), 123.8, 121.4, 116.5 (d, *J* = 21.9 Hz), 52.9, 52.2, 49.7, 36.8; ESI-HRMS calcd for C_18_H_17_FN_2_NaO_5_ (M + Na)^+^ 383.1014, found 383.1011; [α]^20^_D_ + 4.5° (*c* 0.2, MeOH).

**27** was synthesised from **27e** (70 mg, 0.19 mmol) according to the general ester hydrolysis procedure to give 57 mg (88%) of a pale yellow solid: *t*_*R*_ = 9.77 min (HPLC); ^1^H NMR (400 MHz, Acetone-*d*_*6*_) δ 11.25 (br s, 2H), 9.27–9.16 (m, 1H), 8.35–8.21 (m, 2H), 8.21–8.07 (m, 3H), 7.34–7.22 (m, 2H), 5.13–5.03 (m, 1H), 3.19–3.01 (m, 2H); ^13^C NMR (101 MHz, Acetone-*d*_*6*_) δ 172.6, 172.3, 164.7 (d, *J* = 247.3 Hz), 164.5, 155.6, 150.6, 139.7, 135.5 (d, *J* = 3.2 Hz), 129.9 (d, *J* = 8.5 Hz), 123.7, 121.4, 116.5 (d, *J* = 21.8 Hz), 49.5, 36.5; ESI-HRMS calcd for C_16_H_12_FN_2_O_5_ (M − H)^−^ 331.0736, found 331.0745; [α]^20^_D_ + 19.0° (*c* 0.2, MeOH).

### (4′-Methoxy-[1,1′-biphenyl]-3-carbonyl)-*L*-aspartic acid (28)

Dimethyl (4′-methoxy-[1,1′-biphenyl]-3-carbonyl)-*L*-aspartate (**28a**) was synthesised from (4-methoxyphenyl)boronic acid (43 mg, 0.28 mmol) and **7e** (88 mg, 0.26 mmol) according to the general Suzuki coupling procedure. Purification by flash column chromatography (EtOAc:petroleum ether, 1:1) gave 72 mg (75%) of a pale yellow sticky oil: Rf = 0.29 (EtOAc:petroleum ether, 1:1); ^1^H NMR (400 MHz, Acetone-*d*_*6*_) δ 8.20–8.14 (m, 1H), 8.13–8.09 (m, 1H), 7.85–7.76 (m, 2H), 7.67–7.60 (m, 2H), 7.57–7.50 (m, 1H), 7.08–7.02 (m, 2H), 5.11–5.01 (m, 1H), 3.85 (s, 3H), 3.71 (s, 3H), 3.66 (s, 3H), 3.09–2.90 (m, 2H); ^13^C NMR (101 MHz, Acetone-*d*_*6*_) δ 172.1, 171.6, 167.3, 160.7, 141.9, 135.7, 133.4, 130.4, 129.9, 129.0, 126.5, 126.2, 115.3, 55.7, 52.7, 52.1, 50.5, 36.7; ESI-HRMS calcd for C_20_H_21_NNaO_6_ (M + Na)^+^ 394.1261, found 394.1268; [α]^20^_D_ − 23.5° (*c* 0.2, MeOH).

**28** was synthesised from **28e** (64 mg, 0.17 mmol) according to the general ester hydrolysis procedure to give 57 mg (97%) of a pale yellow solid: *t*_*R*_ = 9.92 min (HPLC); ^1^H NMR (400 MHz, Acetone-*d*_*6*_) δ 11.15 (br s, 2H), 8.16–8.08 (m, 2H), 7.87–7.82 (m, 1H), 7.82–7.76 (m, 1H), 7.68–7.61 (m, 2H), 7.56–7.50 (m, 1H), 7.09–7.01 (m, 2H), 5.12–5.02 (m, 1H), 3.85 (s, 3H), 3.13–2.95 (m, 2H); ^13^C NMR (101 MHz, Acetone-*d*_*6*_) δ 172.5, 172.3, 167.5, 160.7, 141.9, 135.8, 133.4, 130.3, 129.9, 129.0, 126.4, 126.2, 115.3, 55.7, 50.3, 36.5; ESI-HRMS calcd for C_18_H_16_NO_6_ (M − H)^−^ 342.0983, found 342.0989; [α]^20^_D_ − 1.5° (*c* 0.2, MeOH).

### (6-(4-Methoxyphenyl)picolinoyl)-*L*-aspartic acid (29)

Dimethyl (6-(4-methoxyphenyl)picolinoyl)-*L*-aspartate (**29e**) was synthesised from (4-methoxyphenyl)boronic acid (42 mg, 0.28 mmol) and dimethyl (6-bromopicolinoyl)-*L*-aspartate (86 mg, 0.25 mmol) according to the general Suzuki coupling procedure. Purification by flash column chromatography (EtOAc:petroleum ether, 1:1) gave 63 mg (67%) of a milky sticky oil: Rf = 0.25 (EtOAc:petroleum ether, 1:1); ^1^H NMR (400 MHz, Acetone-*d*_*6*_) δ 9.20–9.11 (m, 1H), 8.20–8.13 (m, 2H), 8.11–7.98 (m, 3H), 7.12–7.04 (m, 2H), 5.12–5.04 (m, 1H), 3.89 (s, 3H), 3.74 (s, 3H), 3.69 (s, 3H), 3.13–3.01 (m, 2H); ^13^C NMR (101 MHz, Acetone-*d*_*6*_) δ 171.94, 171.93, 164.6, 162.2, 156.5, 150.3, 139.4, 131.4, 129.2, 123.1, 120.6, 115.1, 55.8, 52.8, 52.2, 49.7, 36.8; ESI-HRMS calcd for C_19_H_20_N_2_NaO_6_ (M + Na)^+^ 395.1214, found 395.1217; [α]^20^_D_ + 4.0° (*c* 0.2, MeOH).

**29** was synthesised from **29e** (54 mg, 0.14 mmol) according to the general ester hydrolysis procedure to give 48 mg (96%) of a pale yellow foam: *t*_*R*_ = 9.69 min (HPLC); ^1^H NMR (400 MHz, Acetone-*d*_*6*_) δ 11.26 (br s, 2H), 9.29–9.16 (m, 1H), 8.22–8.13 (m, 2H), 8.11–7.99 (m, 3H), 7.12–7.00 (m, 2H), 5.13–5.01 (m, 1H), 3.88 (s, 3H), 3.24–3.01 (m, 2H); ^13^C NMR (101 MHz, Acetone-*d*_*6*_) δ 172.7, 172.4, 164.7, 162.1, 156.4, 150.4, 139.4, 131.5, 129.1, 123.0, 120.6, 115.1, 55.8, 49.4, 36.6; ESI-HRMS calcd for C_17_H_15_N_2_O_6_ (M − H)^−^ 343.0936, found 343.0932; [α]^20^_D_ + 18.0° (*c* 0.2, MeOH).

### (4′-(Trifluoromethoxy)-[1,1′-biphenyl]-3-carbonyl)-*L*-aspartic acid (30)

Dimethyl (4′-(trifluoromethoxy)-[1,1′-biphenyl]-3-carbonyl)-*L*-aspartate (**30e**) was synthesised from (4-(trifluoromethoxy)phenyl)boronic acid (58 mg, 0.28 mmol) and **7e** (87 mg, 0.25 mmol) according to the general Suzuki coupling procedure. Purification by flash column chromatography (EtOAc:petroleum ether, 2:3) gave 77 mg (72%) of a pale yellow solid: Rf = 0.40 (EtOAc:petroleum ether, 1:1); ^1^H NMR (400 MHz, Acetone-*d*_*6*_) δ 8.23–8.18 (m, 1H), 8.18–8.15 (m, 1H), 7.94–7.90 (m, 1H), 7.89–7.85 (m, 1H), 7.85–7.80 (m, 2H), 7.63–7.57 (m, 1H), 7.49–7.43 (m, 2H), 5.11–5.02 (m, 1H), 3.72 (s, 3H), 3.66 (s, 3H), 3.09–2.91 (m, 2H); ^13^C NMR (101 MHz, Acetone-*d*_*6*_) δ 172.0, 171.6, 167.1, 140.7, 140.4, 135.9, 131.0, 130.1, 129.7, 127.6, 126.8, 122.4, 52.7, 52.1, 50.5, 36.7; ESI-HRMS calcd for C_20_H_18_F_3_NNaO_6_ (M + Na)^+^ 448.0978, found 448.0981; [α]^20^_D_ − 22.5° (*c* 0.2, MeOH).

**30** was synthesised from **30e** (68 mg, 0.16 mmol) according to the general ester hydrolysis procedure to give 60 mg (94%) of a white solid: *t*_*R*_ = 11.38 min (HPLC); ^1^H NMR (400 MHz, Acetone-*d*_*6*_) δ 11.21 (br s, 2H), 8.21–8.18 (m, 1H), 8.18–8.11 (m, 1H), 7.97–7.90 (m, 1H), 7.90–7.80 (m, 3H), 7.64–7.57 (m, 1H), 7.49–7.42 (m, 2H), 5.12–5.02 (m, 1H), 3.11–2.94 (m, 2H); ^13^C NMR (101 MHz, Acetone-*d*_*6*_) δ 172.5, 172.3, 167.2, 149.7, 140.7, 140.4, 136.1, 130.9, 130.1, 129.7, 127.6, 126.8, 122.4, 50.4, 36.5; ESI-HRMS calcd for C_18_H_13_F_3_NO_6_ (M − H)^−^ 396.0700, found 396.0716; [α]^20^_D_ − 1.0° (*c* 0.2, MeOH).

### (6-(4-(Trifluoromethoxy)phenyl)picolinoyl)-*L*-aspartic acid (31)

Dimethyl (6-(4-(trifluoromethoxy)phenyl)picolinoyl)-*L*-aspartate (**31e**) was synthesised from (4-(trifluoromethoxy)phenyl)boronic acid (60 mg, 0.29 mmol) and dimethyl (6-bromopicolinoyl)-*L*-aspartate (86 mg, 0.25 mmol) according to the general Suzuki coupling procedure. Purification by flash column chromatography (EtOAc:petroleum ether, 1:2) gave 76 mg (72%) of a milky sticky oil: Rf = 0.38 (EtOAc:petroleum ether, 1:1); ^1^H NMR (400 MHz, Acetone-*d*_*6*_) δ 9.19–9.11 (m, 1H), 8.36–8.31 (m, 2H), 8.23–8.18 (m, 1H), 8.15–8.11 (m, 2H), 7.53–7.47 (m, 2H), 5.13–5.06 (m, 1H), 3.74 (s, 3H), 3.69 (s, 3H), 3.10–3.04 (m, 2H); ^13^C NMR (101 MHz, Acetone-*d*_*6*_) δ 172.0, 171.9, 164.4, 155.3, 151.0, 150.6, 139.9, 138.2, 129.7, 124.2, 122.2, 121.9, 52.9, 52.2, 49.8, 36.7; ESI-HRMS calcd for C_19_H_17_F_3_N_2_NaO_6_ (M + Na)^+^ 449.0931, found 449.0949; [α]^20^_D_ + 2.0° (*c* 0.2, MeOH).

**31** was synthesised from **31e** (65 mg, 0.15 mmol) according to the general ester hydrolysis procedure to give 58 mg (95%) of a white solid: *t*_*R*_ = 10.98 min (HPLC); ^1^H NMR (400 MHz, Acetone-*d*_*6*_) δ 11.14 (br s, 2H), 9.25–9.18 (m, 1H), 8.37–8.30 (m, 2H), 8.24–8.17 (m, 1H), 8.17–8.10 (m, 2H), 7.51–7.45 (m, 2H), 5.12–5.03 (m, 1H), 3.21–3.00 (m, 2H); ^13^C NMR (101 MHz, Acetone-*d*_*6*_) δ 172.6, 172.3, 164.4, 155.2, 151.0, 150.7, 139.9, 138.2, 129.7, 124.0, 122.1, 121.8, 49.5, 49.4, 36.5; ESI-HRMS calcd for C_17_H_12_F_3_N_2_O_6_ (M − H)^−^ 397.0653, found 397.0665; [α]^20^_D_ + 14.0° (*c* 0.2, MeOH).

### (6-(4-(2,2,2-Trifluoroethoxy)phenyl)picolinoyl)-*L*-aspartic acid (32)

Diethyl (6-chloropicolinoyl)-*L*-aspartate (**32a**) was synthesised from 6-chloropicolinic acid (615 mg, 3.90 mmol) and diethyl *L*-aspartate hydrochloride (679 mg, 3.01 mmol) according to the general amide coupling procedure. Purification by flash column chromatography (EtOAc:petroleum ether, 1:3) gave 840 mg (85%) of an orange oil: Rf = 0.31 (EtOAc:petroleum ether, 1:1); ^1^H NMR (400 MHz, Acetone-*d*_*6*_) δ 8.73 (d, *J* = 7.5 Hz, 1H), 8.13–8.05 (m, 2H), 7.70 (dd, *J* = 7.4, 1.4 Hz, 1H), 5.07–4.99 (m, 1H), 4.28–4.07 (m, 4H), 3.12–2.96 (m, 2H), 1.24 (t, *J* = 7.1 Hz, 3H), 1.23 (t, *J* = 7.1 Hz, 3H); ^13^C NMR (101 MHz, Acetone-*d*_*6*_) δ 171.2, 171.1, 163.1, 151.2, 150.7, 141.9, 128.3, 122.1, 62.1, 61.3, 49.9, 36.9, 14.5, 14.4.

A Schlenck flask under an argon atmosphere was charged with Pd-XPhos-G4 (8 mg, 0.01 mmol), XPhos (9 mg, 0.02 mmol), diboronic acid (181 mg, 2.02 mmol), and KOAc (198 mg, 2.02 mmol). The flask was evacuated and backfilled with argon, before addition of degassed EtOH (7 mL) and 1-chloro-4-(2,2,2-trifluoroethoxy)benzene (141 mg, 0.67 mmol, prepared according to^21^). The flask was evacuated and backfilled with argon and stirred at 80 °C until the reaction turned yellow (30 min). Degassed aqueous K_2_CO_3_ (1.8 M, 1.1 mL) and **32a** (222 mg, 0.67 mmol) dissolved in degassed EtOH (1 mL) were added, and the flask was once again evacuated and backfilled with argon and stirred at 80 °C for 16 hours. After completion, the reaction was cooled to rt, diluted with water and extracted with EtOAc (x3). The organic phases were combined, washed with brine, dried over Na_2_SO_4_ and concentrated *in vacuo*. The residue was purified by flash column chromatography (EtOAc:petroleum ether:AcOH, 1:2:0→99:0:1) to give 73 mg (25%) of (*S*)-4-ethoxy-4-oxo-2-(6-(4-(2,2,2-trifluoroethoxy)phenyl)picolinamido)butanoic acid (**32b**) as a brown sticky solid: Rf = 0.22 (EtOAc + 1% AcOH); ^1^H NMR (400 MHz, Acetone-*d*_*6*_) δ 9.16 (d, *J* = 8.5 Hz, 1H), 8.25–8.20 (m, 2H), 8.15–8.09 (m, 1H), 8.09–8.03 (m, 2H), 7.25–7.17 (m, 2H), 5.06 (dt, *J* = 8.5, 5.3 Hz, 1H), 4.77 (q, *J* = 8.6 Hz, 2H), 4.15 (q, *J* = 7.1 Hz, 2H), 3.07 (qd, *J* = 16.7, 5.3 Hz, 2H), 1.22 (t, *J* = 7.1 Hz, 3H); ^13^C NMR (101 MHz, Acetone-*d*_*6*_) δ 172.4, 172.0, 171.5, 164.6, 159.6, 156.0, 150.4, 139.6, 133.2, 129.3, 122.1 (d, *J* = 238.1 Hz), 116.1, 66.2 (d, *J* = 35.1 Hz), 61.3, 49.6, 37.1, 14.5; ESI-MS *m/z* 439.1 (M − H)^−^; [α]^20^_D_ − 0.5° (*c* 0.2, MeOH).

**32** was synthesised from **32b** (40 mg, 0.09 mmol) according to the general ester hydrolysis procedure to give 29 mg (77%) of a white solid: *t*_*R*_ = 10.84 min (HPLC); ^1^H NMR (400 MHz, Acetone-*d*_*6*_) δ 11.24 (br s, 2H), 9.21 (d, *J* = 8.4 Hz, 1H), 8.26–8.18 (m, 2H), 8.11 (dd, *J* = 8.7, 4.2 Hz, 1H), 8.09–8.02 (m, 2H), 7.26–7.14 (m, 2H), 5.11–5.01 (m, 1H), 4.77 (q, *J* = 8.6 Hz, 2H), 3.21–3.00 (m, 2H); ^13^C NMR (101 MHz, Acetone-*d*_*6*_) δ 172.6, 172.4, 164.6, 159.6, 156.0, 150.5, 139.5, 133.2, 129.4, 124.9 (q, *J* = 276.5 Hz), 123.3, 121.0, 116.1, 66.2 (q, *J* = 35.1 Hz), 49.5, 36.6; ESI-HRMS calcd for C_18_H_14_F_3_N_2_O_6_ (M − H)^−^ 411.0809, found 411.0812; [α]^20^_D_ + 29.9° (*c* 0.2, MeOH).

### (6-(4-Propoxyphenyl)picolinoyl)-*L*-aspartic acid (33)

1-Bromo-4-propoxybenzene (**33a**) was synthesised from 4-bromophenol (250 mg, 1.44 mmol) and 1-iodopropane (0.45 mL, 4.61 mmol) according to the general alkylation procedure. Purification by flash column chromatography (petroleum ether) gave 248 mg (80%) of a clear oil: Rf = 0.62 (EtOAc:petroleum ether, 1:6); ^1^H NMR (400 MHz, Acetone-*d*_*6*_) δ 7.44–7.38 (m, 2H), 6.92–6.86 (m, 2H), 3.94 (t, *J* = 6.5 Hz, 2H), 1.83–1.70 (m, 2H), 1.01 (t, *J* = 7.4 Hz, 3H); ^13^C NMR (101 MHz, Acetone-*d*_*6*_) δ 159.5, 133.0, 117.5, 112.8, 70.4, 23.2, 10.7.

A Schlenck flask under an argon atmosphere was charged with Pd-XPhos-G4 (4 mg, 1 mol%), XPhos (6 mg, 2 mol%), diboronic acid (135 mg, 1.50 mmol), and KOAc (147 mg, 1.50 mmol). The flask was evacuated and backfilled with argon, before addition of degassed EtOH (2.5 mL) and **33a** (114 mg, 0.53 mmol). The flask was evacuated and backfilled with argon and stirred at 80 °C until the reaction turned orange (80 min). Degassed aqueous K_2_CO_3_ (1.8 M, 0.85 mL) and dimethyl (6-chloropicolinoyl)-*L*-aspartate (151 mg, 0.50 mmol) dissolved in degassed EtOH (0.25 mL) were added, and the flask was once again evacuated and backfilled with argon and stirred at 80 °C for 3 hours. After completion, the reaction was cooled to rt, diluted with water and extracted with EtOAc (x3). The organic phases were combined, washed with brine, dried over Na_2_SO_4_, and concentrated *in vacuo*. The residue was purified by flash column chromatography to give a mixture of methyl and ethyl esters of (6-(4-propoxyphenyl)picolinoyl)-*L*-aspartic acid after trans-esterification with the reaction solvent. The crude was therefore used directly in the hydrolysis following the general procedure to give 27 mg (15% over two steps) of **33** as a sticky pale yellow foam: *t*_*R*_ = 11.09 min (HPLC); ^1^H NMR (400 MHz, Acetone-*d*_*6*_) δ 10.89 (br s, 2H), 9.22 (d, *J* = 8.4 Hz, 1H), 8.19–8.10 (m, 2H), 8.10–7.99 (m, 3H), 7.09–7.01 (m, 2H), 5.06 (dt, *J* = 8.6, 5.1 Hz, 1H), 4.04 (t, *J* = 6.5 Hz, 2H), 3.20–2.97 (m, 2H), 1.88–1.72 (m, 2H), 1.04 (t, *J* = 7.4 Hz, 3H); ^13^C NMR (101 MHz, Acetone-*d*_*6*_) δ 172.6, 172.4, 164.7, 161.6, 156.5, 150.4, 139.4, 131.3, 129.1, 123.0, 120.5, 115.6, 70.3, 49.4, 36.6, 23.3, 10.7; ESI-HRMS calcd for C_19_H_19_N_2_O_6_ (M − H)^−^ 371.1249, found 371.1240; [α]^20^_D_ + 49.5° (*c* 0.2, MeOH).

### Kinetic aqueous solubility

Duplicates of a 200 μM solution of the dicarboxylic acids were prepared from 0.01 M PBS_7.4_ and a 10 mM stock solution in DMSO. The samples were incubated in an Eppendorf® Thermomixer (25 °C, 800 rpm) for 24 h. Afterwards, the samples were centrifuged for 5 min at 11,000 rpm and the supernatant was filtered (0.45 μm PTFE membrane) before analysis by HPLC. The solubility was calculated based on a concentration-absorption curve.

### Chemical stability

Triplicates of a 50 μM solution of the test compounds were prepared from 0.01 M PBS_7.4_ and a 10 mM stock solution in DMSO. The samples were incubated in an Eppendorf® Thermomixer (37 °C, 650 rpm). The samples were briefly vortexed and 50 μL aliquots were withdrawn at the time points 0 h, 24 h, 48 h etc. and analysed immediately on UPLC. The chemical stability in PBS_7.4_ was determined at every time point in percentage relative to the 0 h time point.

### Stability in simulated gastric and intestinal fluids

FaSSIF, FeSSGF and FaSSGF were prepared in accordance to the manufacturers procedure, Biorelevant.com. Triplicates of a 50 μM solution of the test compounds were prepared from a 10 mM stock solution in DMSO diluted with FaSSGF/FaSSIF/FeSSIF. The samples were incubated in an Eppendorf® Thermomixer (37 °C, 650 rpm). Samples were withdrawn at 0 min and 120 min, centrifuged (10 min at 10,000 rpm) and the supernatant analysed by HPLC/UPLC. The stability was calculated based on peak area of a 0 point sample.

### logD_7.4_ determination

A glass vial with screw cap (8 mL) was charged with test compound (40 μL, 10 mM in DMSO), PBS_7.4_ (0.01 M, 1980 μL), and 1-octanol (1980 μL). The vial was capped and sealed with parafilm and shaken at 700 rpm using an IKA® KS 125 basic shaker for 24 h at room temperature. The parafilm was removed and the sample was allowed to equilibrate for 1 h before analysis. 100 μL of the octanol phase was withdrawn and diluted 1:10 with MeOH(+0.1% formic acid)/MilliQ water (4:1, v/v) and analysed by HPLC/UPLC. The interface was removed and the PBS_7.4_ phase analysed directly by HPLC/UPLC. All analysis was performed in duplicates and logD values were calculated from the peak areas (mAU*min) and adjusted for difference in injection volume and concentration-absorption effects from the solvents, using two calibration points per compound per solvent, and dilution of the octanol phase. All compounds were analysed in triplicates.

### Metabolic stability

Microsomal stability was studied in mouse liver microsomes (0.5 mg/mL) at a final test compound concentration of 1 μM and performed in triplicates in accordance to the published protocol^22^. In short: Prewarmed (37 °C) 0.1 M PBS_7.4,_ 10 mM NADPH in PBS_7.4_ and test compound (1 mM in DMSO) were added to an Eppendorf^®^ Tube. The samples were incubated for 5 min at 37 °C before addition of newly thawned microsomes. The samples were mixed by gentle vortexing and incubated for 1 h at 37 °C, 300 rpm in an Eppendorf® Thermomixer. Samples were quenched by addition of ice-cold MeOH/MeCN (1:1) and centrifuged for 5 min at 10,000 g. The supernatant was transferred to HPLC vials and stored in the freezer until analysis by HPLC/UPLC. The metabolic stability was calculated based on a 0 min sample. All compounds were analysed in triplicates.

### Molecular biology, cell culture, and transfection

Receptor constructs for mSUCNR1 and hSUCNR1 were bought from Origene and cloned into the eukaryotic expression vector pCMV-Tag(2B) (Stratagene).

HEK-293 cells were cultured in Dulbecco’s modified Eagle’s medium 1885 (DMEM) supplemented with 10% fetal calf serum, 100 units/mL penicillin, and 100 μg/mL streptomycin. Transient transfection of the HEK-293 cells was done with Lipofectamine-2000 according to manufacturer’s protocol. Cells were supplemented with fresh medium after 5 h.

### IP3 turnover assay

96-well plates were coated with poly-D-lysine and HEK-293 cells were plated (35.000 cells/well). Cells were transfected for 5 h the following day and subsequently incubated O/N with 0.5 μCi/mL myo[3H]inositol (Perkin Elmer) in 100 μL growth medium. On day 3 cells were washed with 200 µL HBSS/well (Gibco, Life Technologies) followed by a pre-incubation (30 min, 37 °C) with 100 µL HBSS supplemented with 10 mM LiCl. Cells were stimulated with ligand (120 min, 37 °C) and lysed with 50 µL 10 mM formic acid (30 min on ice). In a white 96 well plate 20 µL cell extracts and 80 µL 1:8 diluted YSi scintillation beads (Perkin Elmer) were mixed. The plate was spun down, and a Packard Top Count NXT counter recorded light emission (scintillation) after an 8 h delay.

### Automated Ligand-guided Backbone Ensemble Receptor Optimization protocol (ALiBERO)

ALiBERO is an iterative sampling-selection protocol for receptor optimisation that relies on the use of ligand information for selecting the best-performing receptor conformations^19^. Homology models of the mouse SUCNR1 receptor were constructed according to Trauelsen *et al*.^4^ and loaded into ICM (Molsoft L.L.C., San Diego, CA, USA). The structure was converted into an ICM object, thereby assigning protein atom types, optimising hydrogens and His, Pro, Asn, Gly and Cys side-chain conformations. The explored chemical compounds were used as a training set by dividing all compounds into an active (EC_50_ ≤ 10 µM) and an inactive (EC_50_ > 10 µM) group, consisting of 25 compounds each. For a list of all compounds used in the optimisation protocol, see Table S1. ALiBERO was performed using the prepared receptor structure and ligand training set as input. Binding site residues were manually selected based on proximity to the position of MRS2500 in the superimposed structure of the P_2_Y_1_ receptor (PDB 4XNW). 100 elastic network normal mode analysis derived conformers were built in order to recreate backbone and side-chain flexibility (T = 300 K). Next, a flexible-ligand static-receptor small-scale virtual screening was performed on each of the receptor conformers, from which several pockets were selected for the following generation. The ligand and decoy molecules were docked into mSUCNR1, represented as pre-calculated potential grids and then sorted according to their ICM VLS scores. The maximum number of complementary pockets for each generation was set to 5 with a maximum of 10 generations. Receptor models were selected based on their combined screening performance, as determined by the normalised square root area under the curve, NSQ_AUC. After each round of virtual screening, an all-atom Monte Carlo side-chain refinement was performed to account for induced-by-ligand changes. NSQ_AUC values were calculated according to Katritch *et al*.^16^.

The optimised model ensemble was validated in a virtual ligand screening with an external test set, consisting of the 25 active compounds used in the ALiBERO optimisation and 1247 decoy molecules that were selected in a similarity search with a Daylight-type fingerprint threshold of T_c_ > 0.6 with the active compound **3** as query molecule and subsequent structural clustering with a T_c_ threshold of 0.15. The test set was docked into the best-performing receptor ensemble from generation 10 of the ALiBERO refinement, using ICM 4D docking. The ROC-plot for the combined performance of the optimised receptor ensemble was computed by taking the best ICL VLS docking score of the receptor models for each docked compound.

### Data availability

The datasets generated and analysed during the current study are available from the corresponding author on reasonable request.

## Electronic supplementary material

Supplementary information

Dataset 1
