# Supplementary material for: Structure-Activity Investigations and Optimisations of Non-metabolite Agonists for the Succinate Receptor 1
Source: Sci Rep. 2018 Jul 3;8:10010. doi: 10.1038/s41598-018-28263-7 (PMC6030209; doi:10.1038/s41598-018-28263-7)
Supplement: Supplementary file 1 — Supplementary information [file 41598_2018_28263_MOESM1_ESM.pdf]

Supporting Information

Structure-Activity Investigations and Optimisations of Non-metabolite Agonists for the Succinate Receptor 1

Elisabeth Rexen Ulven[†]*, *Mette Traulsen*[‡], *Matjaz Brvar*[†], *Michael Lückmann*[§], *Line Ø. Bielefeldt*[†], *Lisa K. I. Jensen*[†], *Thue W. Schwartz*[‡,§], *Thomas M. Frimurer*[‡]

[†]Department of Physics, Chemistry and Pharmacy, University of Southern Denmark, Campusvej 55, 5230 Odense M, Denmark

[‡]NNF Center for Basic Metabolic Research, Section for Metabolic Receptology, Faculty of Health and Medical Sciences, University of Copenhagen, Blegdamsvej 3, 2200 Copenhagen, Denmark

[§]Laboratory for Molecular Pharmacology, Department of Biomedical Research, Faculty of Health and Medical Sciences, University of Copenhagen, Blegdamsvej 3, 2200 Copenhagen, Denmark

Contents

Supplementary Figure S1 S2
Table of compounds for ALiBERO S3
Supplementary Figure S2 S4

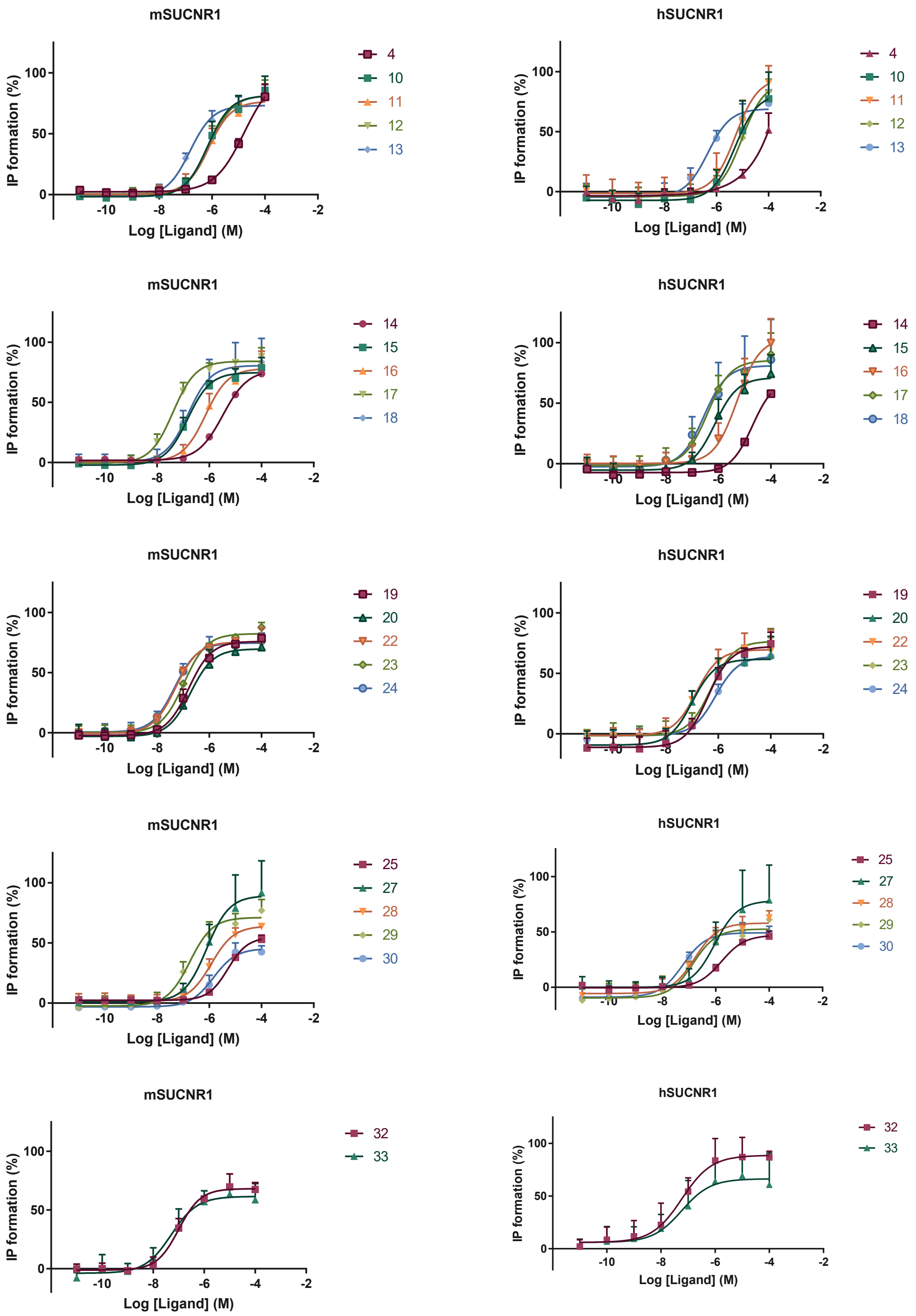

Figure S1: Dose-response curves of active compounds on mSUCNR1 and hSUCNR1.

Table S1: Training set used for ALiBERO optimisation and small-scale virtual ligand screening evaluation of receptor pocket ensembles. Compounds were divided into active (1) and inactive (0) based on an EC_{50} threshold of 10 μM.

Structure (smiles)	Cmpd ID	Active
C(C([O-])=O)[C@H](C([O-])=O)NC(COc1ccc(cc1[Cl])[Cl])=O	(*R*)-184	1
C(C([O-])=O)[C@@H](C([O-])=O)NC(COc1ccc(cc1[Cl])[Cl])=O	(*S*)-184	1
C(C([O-])=O)[C@H](C([O-])=O)NC(c1ccc(c2ccc(cc2)F)o1)=O	(*R*)-130	1
C(C([O-])=O)[C@@H](C([O-])=O)NC(c1ccc(c2ccc(cc2)F)o1)=O	3	1
C(C([O-])=O)[C@@H](C([O-])=O)NC(c1ccc(o1)[Br])=O	4	0
C(C([O-])=O)[C@@H](C([O-])=O)NC(c1ccccc1[Cl])=O	5	0
C(C([O-])=O)[C@@H](C([O-])=O)NC(c1cccc(c1)I)=O	6	0
C(C([O-])=O)[C@@H](C([O-])=O)NC(c1ccc(cc1)[Br])=O	7	0
C(C(N[C@@H](CC([O-])=O)C([O-])=O)=O)c1ccc(cc1)[Br]	8	0
C(C(N[C@@H](CC([O-])=O)C([O-])=O)=O)c1ccc(cc1)c1ccc(cc1)F	9	0
C(C([O-])=O)[C@@H](C([O-])=O)NC(c1ccc(c2ccccc2)o1)=O	10	1
Cc1ccccc1c1ccc(C(N[C@@H](CC([O-])=O)C([O-])=O)=O)o1	11	1
Cc1cccc(c1)c1ccc(C(N[C@@H](CC([O-])=O)C([O-])=O)=O)o1	12	1
Cc1ccc(cc1)c1ccc(C(N[C@@H](CC([O-])=O)C([O-])=O)=O)o1	13	1
C(C([O-])=O)[C@@H](C([O-])=O)NC(c1ccc(c2cccc(CO)c2)o1)=O	14	1
C(C([O-])=O)[C@@H](C([O-])=O)NC(c1ccc(c2ccc(cc2)O)o1)=O	15	1
COc1ccccc1c1ccc(C(N[C@@H](CC([O-])=O)C([O-])=O)=O)o1	16	1
COc1ccc(cc1)c1ccc(C(N[C@@H](CC([O-])=O)C([O-])=O)=O)o1	17	1
C(C([O-])=O)[C@@H](C([O-])=O)NC(c1ccc(c2ccc(cc2)C(F)(F)F)o1)=O	18	1
C(C([O-])=O)[C@@H](C([O-])=O)NC(c1ccc(c2ccc(C#N)cc2)o1)=O	19	1
C(C([O-])=O)[C@@H](C([O-])=O)NC(c1ccc(c2ccc(cc2)OC(F)(F)F)o1)=O	20	1
CCOc1ccc(cc1)c1ccc(C(N[C@@H](CC([O-])=O)C([O-])=O)=O)o1	21	1
CC(C)Oc1ccc(cc1)c1ccc(C(N[C@@H](CC([O-])=O)C([O-])=O)=O)o1	22	1
CC1(COC1)COc1ccc(cc1)c1ccc(C(N[C@@H](CC([O-])=O)C([O-])=O)=O)o1	23	1
CS(CCCOc1ccc(cc1)c1ccc(C(N[C@@H](CC([O-])=O)C([O-])=O)=O)o1)(=O)=O	24	1
C(C([O-])=O)[C@@H](C([O-])=O)NC(c1cccc(c1)c1ccc(cc1)F)=O	25	1
C(C([O-])=O)[C@@H](C([O-])=O)NC(c1ccc(cc1)c1ccc(cc1)F)=O	26	0
C(C([O-])=O)[C@@H](C([O-])=O)NC(c1cccc(c2ccc(cc2)F)n1)=O	27	1
COc1ccc(cc1)c1cccc(c1)C(N[C@@H](CC([O-])=O)C([O-])=O)=O	28	1
COc1ccc(cc1)c1cccc(C(N[C@@H](CC([O-])=O)C([O-])=O)=O)n1	29	1
C(C([O-])=O)[C@@H](C([O-])=O)NC(c1cccc(c1)c1ccc(cc1)OC(F)(F)F)=O	30	1
C(C([O-])=O)[C@@H](C([O-])=O)NC(c1cccc(c2ccc(cc2)OC(F)(F)F)n1)=O	31	1
C(C([O-])=O)[C@H](C([O-])=O)NC(c1ccc(o1)[Br])=O	TUG-1623	0
Cc1c(C(N[C@@H](CC([O-])=O)C([O-])=O)=O)c(c2ccccc2)no1	TUG-1639	0
C(C([O-])=O)[C@@H](C([O-])=O)NC(c1cc2ccccc2[nH]1)=O	TUG-1640	0
C(C([O-])=O)[C@@H](C([O-])=O)NC(c1cccc(c1)Oc1ccccc1)=O	TUG-1641	0
C(C([O-])=O)[C@@H](C([O-])=O)NC(c1ccc(cc1)Oc1ccccc1)=O	TUG-1642	0
C(C([O-])=O)[C@@H](C([O-])=O)NC(c1ccc(nc1)[Cl])=O	TUG-1646	0

C(C([O-])=O)[C@@H](C([O-])=O)NC(c1ccnc(c1)[Cl])=O	TUG-1647	0
C(C([O-])=O)[C@@H](C([O-])=O)NC(c1cccc(n1)[Br])=O	TUG-1648	0
C(C([O-])=O)[C@@H](C([O-])=O)NC(c1cccnc1[Cl])=O	TUG-1649	0
C(C([O-])=O)[C@@H](C([O-])=O)NC(/C=C/c1ccc(cc1[Cl])[Cl])=O	TUG-1652	0
C(C(N[C@@H](CC([O-])=O)C([O-])=O)=O)c1c[nH]c2ccccc12	TUG-1658	0
C(Cc1ccccc1)C(N[C@@H](CC([O-])=O)C([O-])=O)=O	TUG-1674	0
C(C([O-])=O)[C@@H](C([O-])=O)NC(COc1ccc(cc1)OC(F)(F)F)=O	TUG-1675	0
Cc1ccc(cc1)OCC(N[C@@H](CC([O-])=O)C([O-])=O)=O	TUG-1677	0
C(C([O-])=O)[C@@H](C([O-])=O)NC(COc1ccccc1C#N)=O	TUG-1678	0
C(C([O-])=O)[C@@H](C([O-])=O)OC(c1ccc(c2ccc(cc2)F)o1)=O	TUG-1679	0

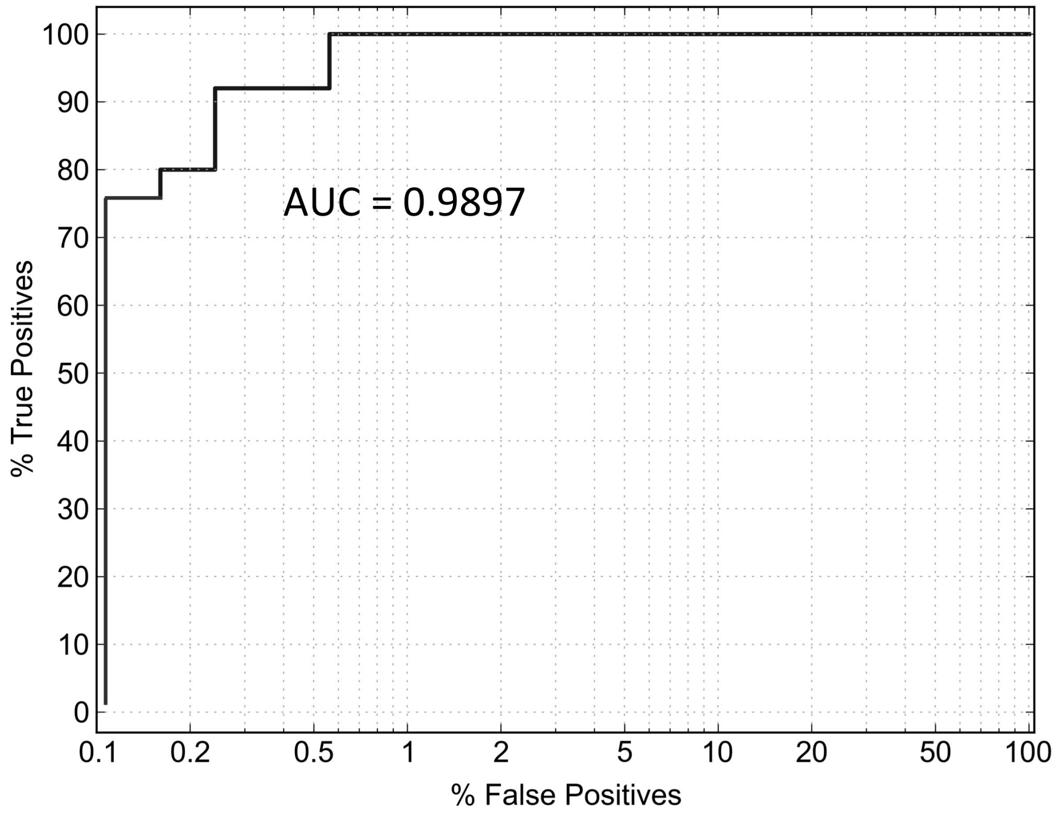

Figure S2: Model validation of the ALiBERO-optimised SUCNR1 models by a large-scale virtual ligand screening. The ROC-curve and area under the curve (AUC) value is shown for the combined performance of the best-performing receptor ensemble of generation 10 of the ALiBERO refinement. A ligand test set consisting of 25 actives and 1247 decoy molecules, that were derived from a similarity search based on the active compound **3** was used.
